# Proteomic Biomarkers for Acute Interstitial Lung Disease in Gefitinib-Treated Japanese Lung Cancer Patients

**DOI:** 10.1371/journal.pone.0022062

**Published:** 2011-07-20

**Authors:** Fredrik Nyberg, Atsushi Ogiwara, Chris G. Harbron, Takao Kawakami, Keiko Nagasaka, Sachiko Takami, Kazuya Wada, Hsiao-Kun Tu, Makiko Otsuji, Yutaka Kyono, Tae Dobashi, Yasuhiko Komatsu, Makoto Kihara, Shingo Akimoto, Ian S. Peers, Marie C. South, Tim Higenbottam, Masahiro Fukuoka, Koichiro Nakata, Yuichiro Ohe, Shoji Kudoh, Ib Groth Clausen, Toshihide Nishimura, György Marko-Varga, Harubumi Kato

**Affiliations:** 1 Global Epidemiology, AstraZeneca R&D, Mölndal, Sweden; 2 Institute of Environmental Medicine, Karolinska Institute, Stockholm, Sweden; 3 Research and Development Division, Medical ProteoScope Company, Tokyo, Japan; 4 Clinical Proteome Center, Tokyo Medical University, Tokyo, Japan; 5 Discovery Statistics, AstraZeneca R&D, Macclesfield, United Kingdom; 6 AstraZeneca in Respiratory Biological Sciences, AstraZeneca R&D, Lund, Sweden; 7 Department of Medical Oncology, Kinki University School of Medicine, Osaka, Japan; 8 Nakata Clinic, Tokyo, Japan; 9 Department of Thoracic Oncology, National Cancer Center Hospital East, Chiba, Japan; 10 Japan Anti-Tuberculosis Association Fukujuji Hospital, Tokyo, Japan; 11 Division of Pulmonary Medicine, Infectious Diseases and Oncology, Department of Internal Medicine, Nippon Medical School, Tokyo, Japan; 12 Respiratory Biological Sciences, AstraZeneca R&D, Lund, Sweden; 13 Department of Surgery, Tokyo Medical University, Tokyo, Japan; 14 Niizashiki Central General Hospital, Saitama, Japan; Cornell University, United States of America

## Abstract

Interstitial lung disease (ILD) events have been reported in Japanese non-small-cell lung cancer (NSCLC) patients receiving EGFR tyrosine kinase inhibitors. We investigated proteomic biomarkers for mechanistic insights and improved prediction of ILD. Blood plasma was collected from 43 gefitinib-treated NSCLC patients developing acute ILD (confirmed by blinded diagnostic review) and 123 randomly selected controls in a nested case-control study within a pharmacoepidemiological cohort study in Japan. We generated ∼7 million tandem mass spectrometry (MS/MS) measurements with extensive quality control and validation, producing one of the largest proteomic lung cancer datasets to date, incorporating rigorous study design, phenotype definition, and evaluation of sample processing. After alignment, scaling, and measurement batch adjustment, we identified 41 peptide peaks representing 29 proteins best predicting ILD. Multivariate peptide, protein, and pathway modeling achieved ILD prediction comparable to previously identified clinical variables; combining the two provided some improvement. The acute phase response pathway was strongly represented (17 of 29 proteins, p = 1.0×10^−25^), suggesting a key role with potential utility as a marker for increased risk of acute ILD events. Validation by Western blotting showed correlation for identified proteins, confirming that robust results can be generated from an MS/MS platform implementing strict quality control.

## Introduction

Interstitial lung disease (ILD) affects the pulmonary parenchyma or alveolar region [1]. When associated with drug treatment, it can present precipitously as acute diffuse alveolar damage (DAD), sometimes with a fatal outcome [2]. Patients often have severe breathlessness and chest radiology shows ‘ground glass’ appearance. No specific treatment is available, but supportive therapy includes oxygen, corticosteroids, or assisted ventilation. Acute ILD events may develop *de novo*, but an existing chronic ILD condition increases the risk considerably [3], as observed in recent studies of patients with idiopathic pulmonary fibrosis (IPF), the most common chronic form [4].

ILD, especially IPF, is a known co-morbidity in patients with non-small-cell lung cancer (NSCLC) [5]. Acute ILD events have been reported with many lung cancer therapies at rates up to ∼10% [6]–[11]. ILD is recognized as more common in Japan than elsewhere, both in the population and among patients with NSCLC [5], [6], [12], [13], although it is unclear why.

EGFR tyrosine kinase inhibitors (TKIs) are an established treatment for advanced NSCLC. Unlike much chemotherapy, they are typically well tolerated and without cytotoxic side effects. The EGFR TKI gefitinib (IRESSA) was approved in 2002 in Japan for treatment of advanced NSCLC. Although some ILD-type events were observed in clinical trials and compassionate clinical use, only after approval did an increasing number of spontaneous reports for ILD appear in Japan as the drug became more widely available.

At that point, better understanding of ILD was urgently needed: baseline incidence on different treatments, risk factors, and the potential association of gefitinib with ILD risk. An independent academic team together with AstraZeneca scientists therefore designed and conducted a cohort and nested case-control pharmacoepidemiological study of ILD in Japanese NSCLC patients treated with either gefitinib or chemotherapy, with clinical results reported previously [3]. As one exploratory sub-study component, patients receiving gefitinib (both subsequent ILD cases and control patients) were sampled for plasma proteomics, with two main objectives: 1) to identify proteomic predictors of ILD that might ultimately be developed into a personalized medicine diagnostic to identify patients at greater risk of ILD; 2) to increase understanding of the mechanisms underlying the development of acute ILD events.

Using a multiple biomarker approach such as proteomics (the simultaneous study of large parts of the human proteome to give a global view of differential expression of proteins in blood or tissue), rather than simply a conventional single biomarker, potentially increases predictive power both through increased robustness deriving from multiple measurements and the opportunity to combine information from multiple biological processes. To support high-quality generation of such information, we combined in a novel way several key study components: robust study design, well-defined phenotypic definitions, careful sample collection procedures, stable advanced liquid chromatography (LC)-tandem mass spectrometry (MS/MS)-based peptide separation and detection methods, statistical analysis incorporating proteomic and clinical information, stringent methods for database protein annotation of detected peptide peaks, and biological interpretation using literature mining software, plus extensive quality control and validation, reported below.

## Results

### Characteristics of the study population

The non-randomized cohort included 3,166 Japanese patients with advanced/recurrent NSCLC who were followed for 12 weeks after initiating gefitinib (n = 1,872 treatment periods) or chemotherapy (n = 2,551). From the gefitinib-treated sub-cohort, 103 suspected ILD cases (79 subsequently confirmed and 24 rejected by the Case Review Board [CRB]), as well as 252 controls, were registered into the case-control study. Proteomics samples for this sub-study were available from 43 confirmed ILD cases, 123 control subjects, and 15 CRB-rejected initially diagnosed ILD cases (Table 1). Clinical characteristics of the cases and controls are described in Table S1.

**Table 1:** Composition of the LC-MS/MS measurement batches for 181 blood plasma samples from Japanese patients with NSCLC.

Batch number	Number of study samples not analyzed in previous batches	Number of analyzed samples^a^	Type of study subject		
			ILD case	Control	Rejected case^b^
1	20	20	3	15	2
2	20	20	5	13	2
3	20	20	6	12	2
4	20	20	3	15	2
5	20	20	6	12	2
6	20	20	5	14	1
7	20	20	6	13	1
8	20	20	3	16	1
9	20	20	6	12	2
10^c^	1^c^	20^c^	3	15	2
11^c^	0^d^	20^d^	6	12	2
Total	181	220	52 (43^e^)	149 (123^e^)	19 (15^e^)

a Each batch also contained 3 experimental control samples in positions 1, 12, and 23 of total batch size of 23.

b Case Review Board (see Materials and Methods) did not confirm clinical ILD diagnosis after blinded diagnostic review.

c 19 samples from batch 1 repeated, 1 new control analyzed.

d All 20 samples from batch 3 repeated.

e Number of unique study subjects.

### Exploratory analysis of LC-MS/MS data generated under quality controlled conditions reveals large batch variation that needs to be controlled in subsequent statistical analyses

#### Quality assessment of sample processing and data generation

After immunoaffinity depletion, remaining serum albumin was <8% for all 181 baseline samples (Table S2). The subsequent tryptic hydrolysis resulted in a remaining undigested protein portion ranging from 3.0% to 32.3% (mean 15.3%) (Table S2). The variation in these processing steps was independent of case/control status (data not shown).

**Figure 1:**
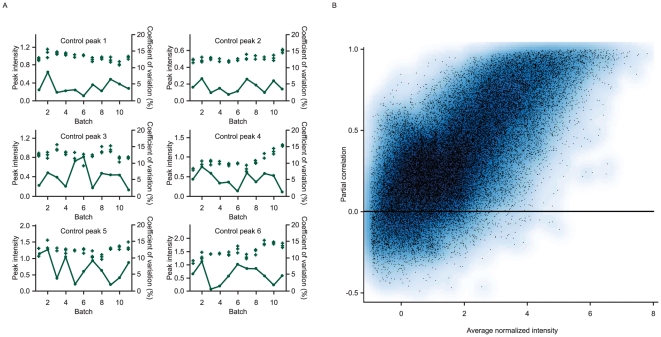
Quality control: reproducibility of control samples and sample duplicates. (A) Reproducibility of 6 control peaks for the 3 standard quality control samples, plotted as ‘+’, in each analysis batch (peak intensity, left axis). The coefficients of variation (%, right axis) between the 3 control samples in each batch are plotted as points joined by a line. (B) Reproducibility of peptide intensities for 39 samples with duplicate analyses in different analysis batches. Partial correlation, after removing between batch differences, plotted against the average normalized intensity for each peptide. Higher intensity peptides show high reproducibility in their intensities between repeated batches.

LC-MS/MS measurements for the 181 individual baseline samples were performed in 11 batches, with 19 and 20 samples from batches 1 and 3 repeated in batches 10 and 11, respectively (Table 1), resulting in 220 discrete proteomics measurements. Four of the 11 batches initially failed the quality control criteria (coefficient of variation [CoV] >20% for any one of the six control peptides among the three within-batch control samples) on the first measurement run, but passed the criteria on repeated measurement. A quality control summary of acceptable batch runs is given in Figure 1A.

Figure 1B shows a plot of partial correlations between the duplicate samples in batches 1 and 10, 3 and 11, after allowing for any batch effect, against the average normalized intensity over the complete sample set for each signal. Peptides with higher average intensities show higher reproducibility between batches as evidenced by generally high partial correlations.

#### Exploratory data analysis of MS signal intensities

**Figure 2:**
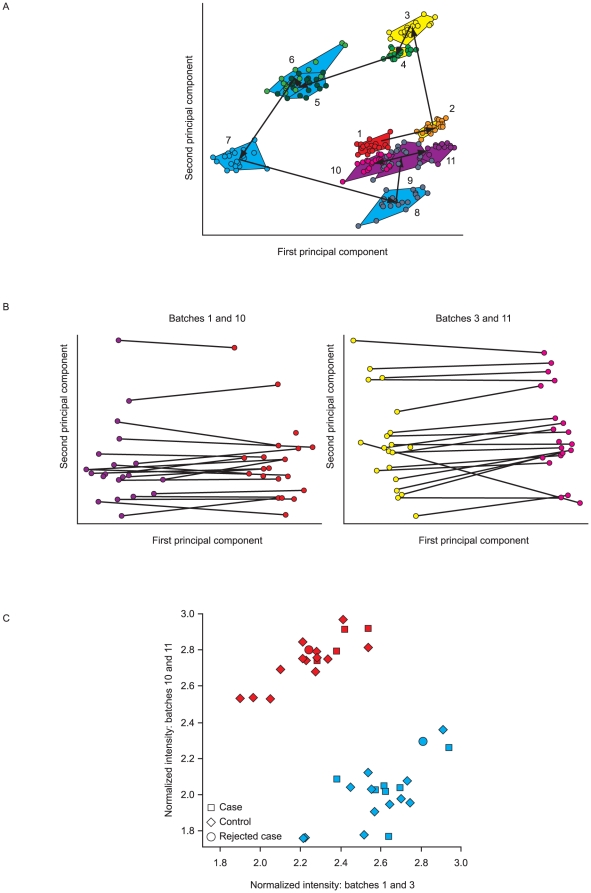
Exploratory data analysis of MS signal intensities using PCA. (A) Plot of first two principal components from PCA analysis of the full proteomic data from all 11 analysis batches (numbered 1–11 in time sequence). Each sample is represented by a single point, with the range of points within each batch being shown by a polygon joining the extreme points in that batch. (B) Plots of the first two principal components for the repeated batches of samples (1 and 10, 3 and 11). Individual samples are represented by a line, connecting the two replicates in different batches. (C) Reproducibility of an example differentially expressed peptide between two duplicate batch runs of proteomic analysis. The intensities of the first and second runs for each replicated sample are plotted against each other. Samples colored by batch (batch 1 repeated as batch 10 – blue; batch 3, repeated as batch 11 – red). Allowing for between-sequence differences there is a good correlation between replicate runs.

We then used a principal component analysis (PCA) to explore the data in order to identify the largest sources of variation. Figure 2A shows a plot of this analysis, with each sample colored according to batch. Measurements from the same batch tend to cluster together, separate from other batches, implying that the largest differences between samples arise from the batch-wise processing.

Figure 2B shows the results of PCA on the pairs of repeated batches (1 and 10, 3 and 11), with duplicate samples joined by a line, plotted against the first two principal components. The lines are generally horizontal and parallel, again suggesting that the largest source of variability or the greatest overall differences in profiles between samples (first principal component) relates to inter-batch variability, and that the ordering of samples on the second principal component, i.e. in the next largest source of variability or overall differences between samples, is in strong agreement between the repeated batches. After allowing for consistent differences between batches, these results thus confirm that inter-sample differences are reproducible with the method used.

Whereas Figure 2B compares results summarized over all measured peptides, Figure 2C shows the repeated run results for an example peptide. Although there are large between-batch differences, within each batch there is high correlation between the intensities for the same subject on replicate runs. Of the 41 differentially expressed peptide peaks used to identify the proteins listed in Table 2, 25 (61%) show a partial correlation after removing the batch effect greater than 0.8 and all show a partial correlation in excess of 0.35.

**Table 2:** List of 29 proteins representing 27 protein identifications from the 41 selected peaks, with pathway assignments according to ingenuity analysis and the validation of ms/ms results using western blots on 12 subjects (6 ILD cases and 6 controls).

Protein name	Acute Phase Response pathway	Correlation between expression levels using MS/MS and Western blot
alpha-1-acid glycoprotein 1	YES	0.717
alpha-1-antitrypsin	YES	0.512
alpha-1B-glycoprotein		
Leucine-rich alpha-2-glycoprotein		
alpha-1-antichymotrypsin	YES	0.744
Antithrombin-III		
Apolipoprotein A-I	YES	0.468
Apolipoprotein B-100		
Apolipoprotein C-III		
Armadillo repeat-containing protein 2		
Complement C3	YES	0.242
Complement C4-A, Complement C4-Ba,b	YES	0.768
Complement component C9	YES	
Plasma kallikrein	YES	
alpha-2-HS-glycoprotein	YES	0.808
Gelsolin		0.873
Hemoglobin alpha		
Hemoglobin beta, Hemoglobin delta^b^		
Haptoglobin	YES	0.859
Haptoglobin-related protein		
Histidine-rich glycoprotein	YES	
Inter-alpha-trypsin inhibitor heavy chain H4	YES	
Retinol binding protein 4	YES	
Serum amyloid P-component	YES	
Serotransferrin	YES	
Transthyretin	YES	
Ig kappa chain V-III region Ti		

a C4 beta chain (common to C4A and C4B).

b Dual identification of 2 closely related proteins from the same protein family.

### Clear differences in peptide and protein patterns between ILD cases and controls

The subsequent analyses aimed to identify peptides and proteins that effectively discriminated between cases and controls, so rejected cases were now excluded. Repeated samples in batches 10 and 11 were excluded, and given the large between-batch differences identified in the exploratory analyses, the control subject measured in batch 10 was also excluded, leaving 43 confirmed ILD cases and 122 controls with one sample measurement each.

#### Identification of discriminating peptides and proteins

**Figure 3:**
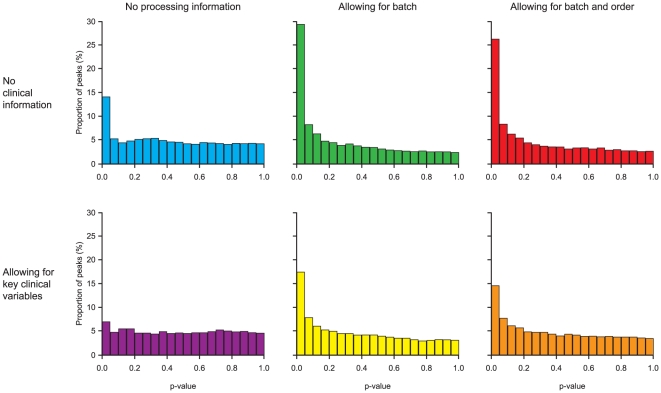
Distribution of significance tests of differential expression between cases and controls for individual peptides. The figure shows the effect on the distribution of p-values for differential expression of including analysis processing information and clinical variables.

Figure 3 shows the results of the univariate (individual peptide) analyses using analysis of covariance (ANCOVA), displayed as histograms of the p-values for the comparison between cases and controls. Allowing for batch as an analysis covariate, to remove inter-batch variation, substantially increases the power of the analysis, identifying approximately twice as many peptides showing statistically significant differential expression at the 5% level. Figures S1 and S2 explore and explain this relationship in more detail. Further accounting for the within-batch order only slightly decreases the number of significant peptides, suggesting that any within-batch order effect is marginal and attempts to model it will not increase power.

On the other hand, allowing for key clinical variables (WHO performance status [PS], smoking history, extent of normal lung coverage on CT scan, and severity of pre-existing ILD) in the analysis consistently reduced the number of peptides being detected as differentially expressed, reflecting that much of the information carried in the most significant peptides is duplicating information carried by the clinical variables (Figure 3). Figure S3 shows an example of this, where higher levels of the peptide, higher performance status score and case status are all associated with each other, and much of the increased peptide intensity of the cases compared with the controls can be explained by their association with higher performance status score, so the peptide intensity is adding much less information when considered in combination with this clinical variable.

Based on the p-value, the top 100 peaks from both the analyses including and excluding clinical variables were identified. These peaks were subsequently restricted to 41 according to the following criteria: 1) normalized LC retention time between 5 and 75 min, and 2) full scan *m/z* value of the precursor ion between 450 and 1,500. Next, peptide identifications included in the 41 peptide peaks were selected using the following criteria: 1) a Mascot ion score more than the identity threshold value given to the individual amino acid sequence of the peptide; and 2) >3 samples with the corresponding peptide identification. This resulted in 45 valid peptide identifications from 28 of the 41 peaks, including two peptides from the spiked lysozyme (Table S3). The plasma-derived 43 peptides represented 27 distinct identifications with 2 dual identifications of closely related proteins, for a total of 29 proteins. These are listed in Table 2, with more detail concerning their identification given in Table S3.

### Acute phase response identified as an important pathway likely to be involved in acute ILD events

**Figure 4:**
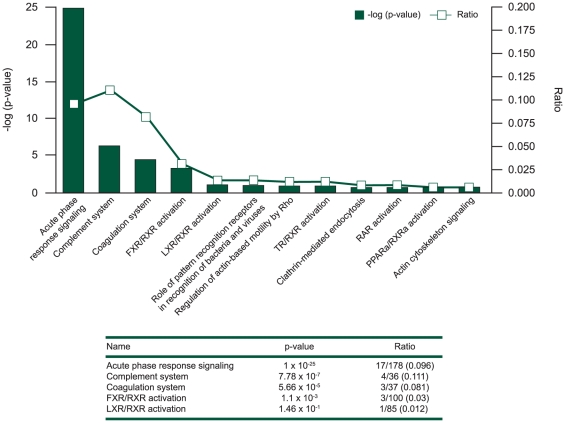
Significant associated pathways with ILD status. The most significant pathways from an analysis linking the identified 29 proteins from the study to curated pathways in the Ingenuity Pathway Analysis system are shown, ordered according to the ratio between the number of protein markers that can be associated with the pathway and the number of proteins in the pathway.

This set of proteins was then used in the biological interpretation analyses, using the Ingenuity Pathway Analysis (IPA) system. The most significant pathway found when overlaying the proteins onto Ingenuity-curated canonical pathways was the acute phase response signaling pathway, with which 17 of the 29 proteins could be associated (p = 1.0×10^−25^). Other pathways showing a high overlap with the list of proteins included the complement and coagulation pathways, but p-values were less significant due to the smaller number of proteins involved (Figure 4).

**Figure 5:**
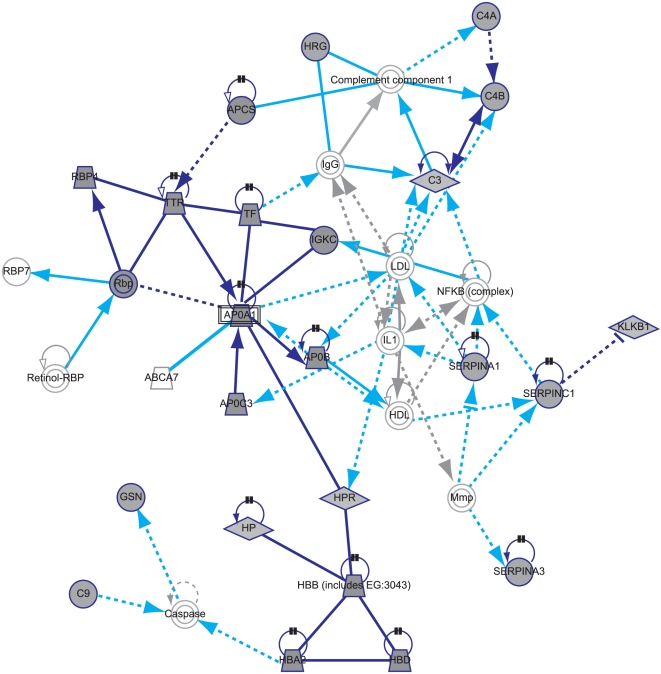
Highest scoring Ingenuity Pathway Analysis network. Highest scoring network generated from entering the identified 29 proteins into the Ingenuity Pathway Analysis system, with proteins identified in the study shaded grey and connecting proteins identified by Ingenuity Pathway Analysis non-shaded. Dark blue shapes and lines  =  proteins identified as predictors in this study and interactions between them. Grey shapes and lines  =  proteins identified by Ingenuity to generate the network and interactions between them. Light blue lines  =  interactions between proteins identified by Ingenuity to generate the network and the proteins identified in the study. Figure S4A shows this figure with the interaction relationships labeled. Proteins identified in the study and included in the network: SERPINA1  =  alpha-1-antitrypsin; SERPINA3  =  alpha-1-antichymotrypsin; SERPINC1  =  antithrombin-III; APOA1  =  apolipoprotein A-I; APOB  =  apolipoprotein B-100; APOC3  =  apolipoprotein C-III; C3  =  complement C3; C4A, C4B  =  complement C4-A; complement C4-B; C9  =  complement component C9; GSN  =  gelsolin; HBA2  =  hemoglobin alpha; HBB, HBD  =  hemoglobin beta/delta; HP  =  haptoglobin; HPR  =  haptoglobin-related protein; HRG  =  histidine-rich glycoprotein; KLKB1  =  plasma kallikrein; IGKC  =  Ig kappa chain V-III region Ti; RBP4, Rbp  =  retinol binding protein 4; APCS  =  serum amyloid P-component; TF  =  serotransferrin; TTR  =  transthyretin. Proteins identified in the study and not included in the network: ORM1  =  alpha-1-acid glycoprotein 1; A1BG  =  alpha-1B-glycoprotein; LRG1  =  leucine-rich alpha-2-glycoprotein; ARMC2  =  armadillo repeat-containing protein 2; AHSG  =  alpha-2-HS-glycoprotein; ITIH4  =  inter-alpha-trypsin inhibitor heavy chain H4.

Entering the 29 proteins into IPA, 5 networks were formed. The most significant network contains 24 of the 29 proteins (Figure 5). Proteins added to the network by the tool to connect the marker proteins include IL1 and NF-κB, suggesting that these proteins could also be involved in generating the observed pattern. Combining the two networks with the highest scores further adds IL1-beta, HNF1A, HNF4A, HNF6 (ONECUT1), and CEBPB as central components (Figure S4).

### Validation of the MS/MS data shows good reproducibility and reasonable agreement with Western blot

Within the MS/MS platform there was strong agreement between replicate runs of the same samples after allowing for batch effects, as described above (Figure 2C).

Validating with another method, Table 2 shows the correlation in intensities derived from the MS/MS and Western blotting (WB) for a selection of 9 proteins. Considering that WB targets the intact protein, whereas the present MS/MS can detect peptides derived from the intact proteins, these 9 proteins show quite a strong level of agreement between the technologies, with 6 of the 9 proteins exhibiting a correlation in excess of 0.7. Scatterplots comparing the MS/MS and WB protein intensities are shown in Figure S5 and the WB images in Figure S6.

### Prediction of ILD using proteins and clinical data

#### Modeling phenotype based on multiple peptide markers

**Figure 6:**
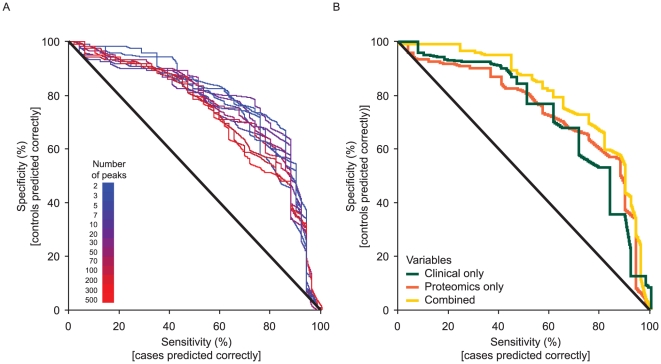
Receiver operating characteristics curve of cross-validated predictions. (A) from peptides, for different number of peptides included in the proteomic prediction model, and (B) from clinical data, proteomic data, and a combination of both clinical and proteomic data.

Figure 6A shows the predictive power based on leave-one-out cross-validation for models built using a range of different numbers of peptides in combination. Substantial improvements on random prediction were obtained from just a few peptides, and increasing the number of peptides further did not substantially improve the model predictions. The predictive power of the model even decreased when using very many peptides.

For robustness, alternative multivariate modeling approaches were compared. Using random forests instead of partial least squares discriminant analysis (PLS-DA) and logistic regression within the modeling framework yielded approximately the same predictive level as evidenced by the area under the curve (details not shown).

A subgroup analysis restricting the set of cases to those with the DAD acute ILD pattern (20 of the 43 cases) was hampered by small sample size and was unable to improve the overall predictive power.

#### Modeling phenotype based on multiple peptide markers and clinical data

Figure 6B compares the predictive power, based on leave-one-out cross validation, for models built on clinical/radiological data alone using a logistic regression, peptide data alone, and a combination. Both data types alone provided similar prediction. Some improvement was obtained by combining the two data types, but it was far less than additive. This is consistent with the results from the analysis of individual peptides, suggesting that the discriminating peptides partly carry information also available from the clinical/radiological variables.

#### Modeling phenotypes based on proteins and clinical data

**Figure 7:**
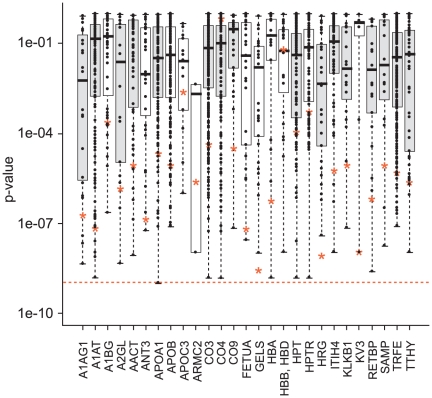
Significance levels from the proteins, constituent peptides, and acute phase pathway intensities. p-values for the proteins are shown by red stars, p-values for individual peptides are shown by points, and the distribution of these for each protein is shown by a boxplot. In each boxplot, the upper and lower sides of the box represent the higher and lower quartile values (Q3 and Q1), respectively. The black bar in each box represents the median value. The p-value for the acute phase pathway is represented by the dashed line; boxplots for proteins in the acute phase response pathway are shaded. A1AG1  =  alpha-1-acid glycoprotein 1; A1AT  =  alpha-1-antitrypsin; A1BG  =  alpha-1B-glycoprotein; A2GL  =  leucine-rich alpha-2-glycoprotein; AACT  =  alpha-1-antichymotrypsin; ANT3  =  antithrombin-III; APOA1  =  apolipoprotein A-I; APOB  =  apolipoprotein B-100; APOC3  =  apolipoprotein C-III; ARMC2  =  armadillo repeat-containing protein 2; CO3  =  complement C3; CO4  =  complement C4-A; complement C4-B; CO9  =  complement component C9; FETUA  =  alpha-2-HS-glycoprotein; GELS  =  gelsolin; HBA  =  hemoglobin alpha; HBB, HBD  =  hemoglobin beta/delta; HPT  =  haptoglobin; HPTR  =  haptoglobin-related protein; HRG  =  histidine-rich glycoprotein; ITIH4  =  inter-alpha-trypsin inhibitor heavy chain H4; KLKB1  =  plasma kallikrein; KV3  =  Ig kappa chain V-III region Ti; RETBP  =  retinol binding protein 4; SAMP  =  serum amyloid P-component; TRFE  =  serotransferrin; TTHY  =  transthyretin.

Figure 7 shows the p-values for distinguishing cases and controls obtained from the proteins (i.e. combined constituent peptide score), all their constituent peptides, and the combined acute phase pathway intensity measure (i.e. the combined score of the 16 included constituent proteins). For most proteins, the estimated protein intensity is more significant than most of the measured peptide intensities associated with that protein, but only improves on the significance of the best peptide for a few proteins. As these results were obtained within the same dataset that was used to identify and select the constituent peptides, some over-fitting may be occurring, and the protein expression intensity incorporating information from many peptides may be a more robust measure to apply in a wider context. The combined acute phase pathway intensity measure shows a more significant response than any of the constituent proteins. A similar picture is obtained when we consider the additional information provided by the peptide, protein and pathway measures on top of the known clinical variables in predicting ILD status (Figure S7).

**Figure 8:**
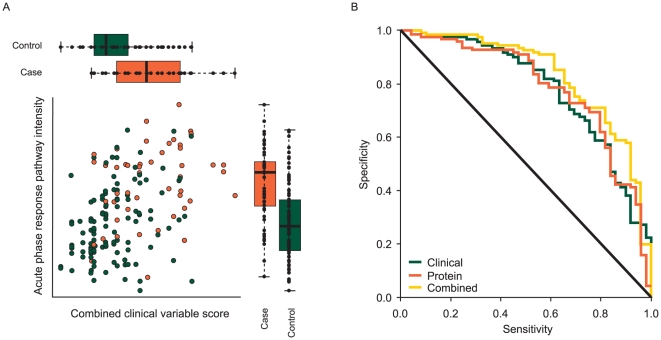
Plots illustrating the relationship between acute phase response pathway intensity score and clinical variable score. (A) Plot of the acute phase response intensity against the combined clinical variable score measuring the likelihood of a subject being a case calculated from a model predicting case-control status based only on the clinical variables WHO PS, smoking history, extent of normal lung coverage on CT scan, and severity of pre-existing ILD, with boxplots comparing the distribution of these measures in cases and controls. In each boxplot, the upper/right and lower/left sides of the box represent the higher and lower quartile values (Q3 and Q1), respectively. The black bar in each box represents the median value. (B) Receiver operating characteristics curve of cross-validated predictions from clinical data, the acute phase response intensity and a combination of the clinical data and acute phase response intensity.

Figure 8A shows the acute phase response pathway intensity plotted against a combined clinical variable score measuring the likelihood of a subject being a case calculated from a logistic regression of case-control status against the clinical variables WHO PS, smoking history, extent of normal lung coverage on CT scan, and severity of pre-existing ILD. This shows both sources of information contributing to predicting ILD outcome, although these two measures are fairly strongly correlated, so that much of the information is duplicated. Figure 8B considers the implications for predicting ILD by showing the receiver operating characteristics (ROC) curves for the clinical variables, the acute phase pathway intensity, and the combination of the two sources of information. This shows comparable levels of predictive power from the clinical variables and acute phase pathway intensities, and some potential benefit from combining them together which, however, is limited, reflecting their correlation.

## Discussion

We present here results from a proteomic analysis applied to a large-scale pharmacoepidemiological study and demonstrate that with considerable attention to study design and experimental procedures throughout the entire process required to generate high-quality data, it is possible to derive valuable knowledge from both a scientific and a diagnostic perspective. However, there are numerous potential sources of data variation and bias in this process. The integrity of all of the steps of the process is critical to generating useful data and failure to ensure high quality in any one of them may compromise the validity and value of the entire study.

### Methodological aspects

#### Study design and sample preparation

We applied careful phenotyping with blinded diagnostic review to ensure an accurate ILD diagnosis, and incorporated measures to ensure that all cases of ILD occurring in the source cohort would be captured. Controls were selected from the actual population generating the cases, ensuring comparability between cases and controls so that contrasts seen may be attributed to case status. Participation rates were high (>90%) and similar for cases and controls [3], making selection bias unlikely. Proteomics samples were obtained after separate informed consent from approximately half of all gefitinib-treated cases and controls. Steps to ensure high-quality proteomic data for our large-scale epidemiological investigation included randomization of the processed samples, careful quality assessment of sample preparations and optimized preparation protocols to ensure stability in all procedures for a large number of samples. We have previously described the general strategy that we decided to use in the study based on a number of experimental pilot phase rounds [14].

#### Experimental measurement batch effects

Two-dimensional polyacrylamide gel electrophoresis is one common conventional technology used for protein analysis in serum/plasma [15], [16]. Currently, LC-MS/MS has become widely accepted for high-resolution proteome-wide profiling from a complex peptide mixture [17]. Recent advances in this methodology including improved stability of peptide separation and detection has enabled comparison of ion intensity between LC-MS/MS profiles [18]. Our proteomics analysis system applied LC-MS/MS after immunoaffinity depletion of the most abundant constituent proteins in blood plasma, and proteolytic enzyme treatment of the depleted plasma sample.

We identified that the LC-MS/MS measuring process has systematic measurement errors, as one might expect, which we took measures to eliminate by introducing batch processing with quality control, designing the order of sample processing to minimize the effects of any systematic errors, and then eliminating batch effects in the statistical analysis. The consideration of batch effects in the statistical analysis appeared to improve the process of detecting discriminating peaks, and we therefore based our final protein identification step on this analysis approach.

#### Alignment algorithms – internal standard-guided Optimal Profile ALignment (i-OPAL)

In order to allow comparative quantification between samples, the sample measurements have to be aligned – i.e. the correspondence of ion signals must be identified. Various methods have been proposed for this, e.g. based on stable isotope labeling [19]–[21], utilizing comparative identification [22], [23], or with direct comparison of the respective peptide ion signals [18]. The i-OPAL method belongs to the last category. In spite of the relatively low accuracy and mass resolution of *m/z* measurement, the MS instrument of ion-trap type allows a long-term stable measurement without any calibration operation [24], [25]. Consequently, the *m/z* values are directly comparable in a large set of samples without further transformations.

### Biological findings and implications

#### Potential biological mechanisms underlying acute ILD events

In our IPA mapping to canonical biological pathways, acute phase proteins came out as the strongest signal, followed by the complement and coagulation pathways. Activation of acute phase response with connection to the complement and coagulation systems have been suggested as key processes in acute ILD events following blood transfusions (transfusion related acute lung injury; TRALI) [26] and in patients with idiopathic thrombocytopenic purpura (ITP) [27]. Acute phase responses can be induced by bronchoscopy with bronchoalveolar lavage [28]. Available evidence strongly suggests that balance between injury and repair is fundamental for regulating injury repair and protecting the lung [29]. In our study, clinical findings suggested that patients had increased risk of acute ILD events early after lung cancer diagnosis, if they had pre-existing chronic ILD, and if their remaining normal lung coverage as assessed on CT scan was low [3], suggesting that factors associated with active or extensive disease processes and/or cancer diagnostic procedures were important. Interestingly, another key protein signal (outside the acute phase pathway) for ILD risk in our study was gelsolin. Gelsolin was recently highlighted by comparative expression profiling and animal experiments as necessary for the development of modeled pulmonary inflammation and fibrosis, and caspase-3 mediated gelsolin fragmentation was shown to be an apoptotic effector mechanism and a marker of lung injury, again emphasizing the balance between injury and repair [30]. Interesting protein connections were also revealed by the IPA-generated networks. For example, CEBPB (NFIL6) is a principal effector of cyclin D1 activity in human cancer and an enhancer of e.g. IL-6 transcription, which plays an important role in the acute phase response [31], [32]. It is important to clarify that IPA-generated networks are not the same as canonical biological pathways, but rather connect different proteins and genes based upon a wide range of interactions reported in the scientific literature.

#### Biomarker validation

Within this study we have validated our conclusions on several different levels. Technical validation by repeating the analysis of the same samples has confirmed the ability of the technology to reproducibly measure the levels of the peptides within the samples. This has been strengthened by the use of alternative technologies to confirm the intensities of key proteins. Together, these two sets of validation data show that the protein intensities derived from this MS/MS analysis are both reproducible and in agreement with those found from other technologies. In combination, they provide strong evidence of the reliability of these data, giving confidence in further interpretation of the results. Biological validation has examined peptides from the same proteins and identified strong levels of correlation, strengthening and helping to confirm our hypotheses. Internally, cross-validation was used as an efficient method for avoiding over-fitting of a multivariate dataset and estimating an error rate of the modeling process whilst still maximizing the use of a limited number of samples. A final level of validation, which has yet to be addressed in this work, would be to validate our conclusions in a completely independently collected set of samples. Given the relative rarity of ILD and the difficult diagnosis, such a dataset remains to be assembled.

#### Biomarker properties

The true practical utility of any molecular diagnostic is not only its ability to make a prediction of outcome, but also its ability to add additional, alternative, and more timely information to assist the physician in treating the patient, at a reasonable cost and effort. With ILD, using clinical and radiological information which may often be easily available, a physician is able to make some assessment of the risk of a patient developing ILD, although this evaluation at present is inexact and difficult to apply consistently. While a patient's proteomic profile appears to provide similar prediction using an alternative method, and may even improve the accuracy of risk assessment when added to clinical/radiological information, that improvement is limited. However, the more objective and possibly more reproducible character of a proteomic measurement might provide advantages over a purely clinical assessment. It may also be noted that the additional value of any component of a risk score as assessed by ROC change is often very small, as has been demonstrated for well-known clinical lab tests such as HDL, HbA1C, and hsCRP in the context of clinically validated Reynolds Risk Score for cardiovascular disease [33], but that the individual components contributing to a score or data compilation used for a clinical decision may nevertheless all contribute to elevating the combined information above the threshold of clinical utility. Nevertheless, from both a medical and a commercial perspective and considering that further validation in independent sets of patients is still required, as well as development of a practical, cost-efficient, timely, and clinically available assay, it is not obvious at this point whether the possible added value justifies further development of the technology as a potential diagnostic.

### Conclusion

This study has identified proteomic markers (peptides) that show a reasonable predictive power for ILD. However, as might be expected, the information they carry appears to overlap partly with that from clinical/radiological data previously shown to predict ILD, including WHO performance status score, smoking history, lung coverage on CT scan, and severity of pre-existing ILD. When combining the two, some gain in predictive power is obtained, although this is limited based on evaluation by ROC curves. Basing the predictive model upon the proteins/pathways identified as interesting shows potential for greater predictive power. In particular, the data suggest that activated acute phase response could be a marker for increased risk of acute ILD events following drug exposure. Whether this is a general mechanism that may be true also for acute ILD exacerbations in other settings, as some recent reports would suggest, is an important question for further research. In any case, our findings again highlight the potential importance of the balance between destruction and repair mechanisms for maintaining a functioning lung. If the results regarding the acute phase pathway can be confirmed, this may lead to a better mechanistic understanding of the basis for ILD events occurring, which would have potentially great future clinical utility as ILD events are an important consideration in the development of many potent new drugs, particularly in the areas of respiratory disease and oncology. In addition, such understanding would allow a more targeted approach to identifying and defining proteomic biomarkers with higher predictive value and clinical utility than was possible in this exploratory study.

## Materials and Methods

### Study design – patients and data collection

This non-randomized cohort study with a nested case-control component was conducted in November 2003 to February 2006 in 50 centers across Japan. Patients with advanced or recurring NSCLC with at least one previous chemotherapy regimen were eligible. Patients and their physicians selected a treatment (gefitinib 250 mg or chemotherapy), and follow-up was extended for up to 12 weeks after treatment initiation. Study design has been described in more detail previously [3]. This proteomics sub-study was performed only with gefitinib-treated patients from the case-control study component.

Patients who developed acute ILD during follow-up were registered to the case-control study nested within the cohort, as clinically diagnosed potential cases. For each potential case, four controls were randomly selected from patients then registered to the cohort who had not developed ILD.

To ensure valid and sensitive ILD diagnosis, the study included 1) an information card describing the symptoms of ILD distributed to all cohort patients; 2) internationally agreed criteria for ILD diagnosis and a diagnostic algorithm developed from an international consensus statement [1]; and 3) an independent CRB of radiologists and clinicians for blinded diagnostic review of all clinically diagnosed potential ILD.

For cases and controls, detailed patient data were collected covering NSCLC treatment, demography, cancer histology, clinical staging, WHO PS, smoking, previous cancer treatments, past and current medical history, surgical history, and concomitant medication and therapy. For gefitinib-treated cohort members consenting to the proteomics sub-study, one baseline 6-mL sodium-heparinized blood sample was drawn immediately (1–3 h) after the first gefitinib dose at registration into the cohort. Samples were spun for 10 min at 3,000 rpm and the plasma was stored at –80°C.

Subjects were recruited from 50 centers in Japan, and the Institutional Review Board of each site approved the study and informed consent forms. Written informed consent was required for registration to the study cohort, and separately for registration to the case-control study and for participation in the proteomics study.

### Sample and data processing

The study was performed with quality control procedures at each sample processing, data generation, and data processing step, as described below.

#### Immunoaffinity depletion of serum albumin and IgG from the blood plasma and tryptic hydrolysis of plasma proteins

Depletion of the blood plasma samples was carried out using a dual Albumin and IgG Removal Kit (GE Healthcare UK Ltd, Amersham Place, Buckinghamshire, UK) according to the manufacturer's instructions, with some modifications. An affinity resin of a single production lot was used for the depletion process throughout this investigation. The depletion procedure for the 181 baseline samples was carried out in 18 batches (Table S1). As an experimental control, pooled human plasma (Sigma-Aldrich Inc., St. Louis, MO, USA) was simultaneously subjected to the same experimental procedures. Prior to the depletion the small debris part of the biofluid was removed by filtration. Aliquots (70 µL) of the plasma solution were diluted with 4.0 mL of the suspended gel slurry containing slurry beads with immobilized protein G binding polyclonal antibodies against both human serum albumin and IgG [34]. The sample was incubated on a rotator (5 rpm, 30 min, room temperature) to keep the gel slurry in suspension. Subsequently, the serum albumin/IgG-binding slurry beads were removed from the sample solution by centrifugation (7,000× *g*, 5 min) using a Vivaspin 6 column with polyethersulfone membrane (pore size 0.2 µm; Sartorius AG, Goettingen, Germany). The recovered liquid fraction (approximately 3.3 mL), containing unbound plasma proteins, was subjected to a buffer-exchanging process as follows: the fraction was condensed on a 3,000 molecular weight cutoff membrane of polyethersulfone in a diafiltration vessel (Vivaspin 2 column, Sartorius AG, Goettingen), followed by dilution with excess volume (2 mL) of 50 mM ammonium bicarbonate. This cycle was repeated three times. Finally the resulting solution was condensed to less than 100 µL on the same membrane and adjusted to 200 µL with 50 mM ammonium bicarbonate. The total protein concentration of the depleted solution was measured according to Lowry et al [35], using a DC protein assay kit (Bio-Rad Laboratories Inc., Hercules, CA, USA) with bovine serum albumin as the calibration standard. To confirm the depletion treatment, the concentration of the human serum albumin remaining in the depleted protein solution was measured as follows: An aliquot of the depleted solution was subjected to sodium dodecylsulfate (SDS) polyacrylamide gel electrophoresis (PAGE) [36]. Protein bands on the polyacrylamide gel were stained with a SYPRO Ruby fluorescence dye (Invitrogen Co., Carlsbad, CA, USA), followed by scanning of the gel slab on a LAS-3000 imaging system (FUJIFILM Co., Tokyo, Japan). Finally the fluorescence intensity ratio of the serum albumin band to the all protein bands detected on the gel lane was calculated using a Multi Gauge image analyzing software (FUJIFILM). The samples were then stored at –80°C until use.

#### Tryptic hydrolysis of the plasma proteins

The hydrolysis procedures were carried out in a single batch (Table S1). An aliquot containing 200 µg of the depleted plasma sample was spiked with 250 pmol of egg white lysozyme (Sigma-Aldrich Inc., St Louis, MO, USA) used as a source of exogenous internal standard peptides. Next the samples were denatured by incubating by gentle agitation in 200 µL of 100 mM ammonium bicarbonate containing 25% (v/v) acetonitrile at 37°C for 60 min. The resulting solution was immediately subjected to reductive *S*-carboxyamidomethylation of the sulfhydryl groups of the cysteine residues: incubation by gentle agitation at 37°C for 45 min with addition of 1 µmol of Tris(2-carboxyethyl)phosphine (TCEP) (20 µL of 50 mM solution), followed by the addition of 5 µmol of iodoacetamide (20 µL of 250 mM solution) at room temperature for 60 min in the dark. The Cys-modified proteins were further subjected to tryptic hydrolysis by the addition of 4 µg of porcine trypsin (20 µL of 0.2 mg/mL solution) (Promega Co., Madison, WI, USA) and incubation at 37°C for 16 h. The resulting peptide mixture was stored at –80°C until use.

To measure the degree of hydrolysis, aliquots of the sample solutions before and after the hydrolysis treatment were subjected to SDS PAGE [36], followed by obtaining a fluorescence gel image as described in the previous section. The total fluorescence intensities of the protein bands were compared between both solutions to calculate the protein portion remaining due to incompleteness of the hydrolysis.

#### LC-MS/MS measurement procedures

The peptide mixture was then dissolved in 1.0% v/v trifluoroacetic acid (TFA) aqueous solution with the final peptide concentration of 0.1 mg/mL, and analyzed using an LC-MS/MS system with a Finnigan LTQ linear ion trap mass spectrometer (Thermo Fisher Scientific Inc., Waltham, MA, USA) [25] in a fully automated manner. Briefly, peptide separation was performed with a Paradigm MS4 LC instrument (Michrom BioResources Inc., Auburn, CA, USA) containing a MAGIC C_18_ capillary LC column (0.2 mm id, 50 mm length, 3 µm particle size, and 200 Å pore size; Michrom BioResources). The mobile phase consisted of formic acid, acetonitrile, and water at a volume ratio of 0.1∶2∶98 for mobile phase A, and 0.1∶90∶10 for mobile phase B. The initial flow of 100 µL/min was reduced by a flow splitter to approximately 1 µL/min. 10 µL of the peptide solution, containing 1 µg peptide, was applied using an HTS PAL autoinjector (CTC Analytics AG, Zwingen, Switzerland) onto a Peptide CapTrap column (0.5 mm id, 2.0 mm length, bed volume 0.5 µL; Michrom BioResources) equilibrated with a solution of TFA, acetonitrile, and water at a volume ratio of 0.1∶2∶98. The peptides concentrated and purified on the trap column were injected onto the C_18_ capillary LC column by valve switching. The peptides were continuously eluted at a rate of 1 µL/min on a gradient mode: The initial ratio of 5% of mobile phase B was increased linearly to 40% B during 70 min, followed by the increase to 95% B during the next 5 min. After washing with a non-gradient flow at 95% B, the column was equilibrated again with the solvent of 5% B for the next separation. The total analysis time was 90 min. For gasification of the protonated peptides, the LC effluent was interfaced with an electrospray ionization (ESI) source in a positive ion mode, on a Finnigan LTQ linear ion trap mass spectrometer (Thermo Fisher Scientific) [25]. The ESI used a FortisTip spray emitter (20 µm id, top Teflon-coated; AMR Inc., Tokyo, Japan) directly connected with the outlet of the LC column. The set parameters included a spray voltage of 2.0 kV and a capillary temperature of 200°C. No sheath gas was supplied during the ESI. The other parameters on the ion separation and detection were optimized according to an Autotune function in the mass spectrometer instrument. For MS/MS, protonated peptides in a gas phase were sequentially analyzed by data-dependent scanning mode of a full scan at an *m/z* range of 450 to 2,000 and subsequent product ion scans of the three most intense precursor ions. The data acquisitions were made in a Centroid mode for both scans. The product ion scan was performed under conditions including an intensity threshold of 1×10^3^, 30% normalized collision energy, 2.0 Da isolation *m/z* width, and dynamic exclusion for 30 sec. The ESI-MS/MS operation and data acquisition were carried out on an Xcalibur Revision 1.4 SR1 system controller (Thermo Fisher Scientific).

The LC-MS/MS measurement was continuously performed with alternate injection of the sample solutions and blank solutions for system washing. A single continuous LC-MS/MS measurement batch comprised 23 samples; 20 peptide solutions from patients and 3 experimental control samples of pooled plasma at the initial, middle, and final positions. The analysis order of patient samples was randomized across and within batches, whilst ensuring reasonable balance between batches in case/control status (Table 1).

Measurement variability of the ion intensity within each analysis batch was assessed as the CoV between the three control samples for the relative ion signal area of each of 6 selected common peptides, i.e. the ratio of the total signal area of the differently charged ions shown in Table 3, to the signal area of the most stably detected internal standard peptide (IS1) derived from the spiked lysozyme (Table 3).

**Table 3:** Peptides used as internal standards and for assessment of the ion intensity variations across the LC-MS/MS measurement batches.

	Amino acid sequence (From – To)^a^	Protein name	Retention time, min^b^	Ion *m/z* value^c^ (charge)	Swiss-Prot^d^ accession number
Peptides for assessment of the ion intensity variations					
	EGTC^e^PEAPTDEC^e^KPVK (347–362)	Transferrin	10.8–16.6	910.0 (2+), 607.0 (3+)	P02787
	LRTEGDGVYTLNNEK (117–131)	Haptoglobin	19.8–25.2	1,709.9 (1+), 855.4 (2+), 570.6 (3+)	P00738
	AVGDKLPEC^e^EADDGC^e^PKPPEIAHGYVEHSVR (78–108)	Haptoglobin	27.7–32.5	1,717.9 (2+), 1145.6 (3+), 859.5 (4+)	P00738
	DYVSQFEGSALGK (52–64)	Apolipoprotein A–I	38.0–45.0	1,401.5 (1+), 701.3 (2+)	P02647
	HSTIFENLANKADRDQYELLC^e^LDNTR (226–251)	Transferrin	47.8–53.7	1,569.7 (2+), 1046.8 (3+), 785.4 (4+)	P02787
	TSESGELHGLTTEEEFVEGIYKVEIDTK (69–96)	Transthyretin	58.7–66.0	1,571.7 (2+), 1048.1 (3+), 786.4 (4+)	P02766
					
Internal standard (IS) peptides, two exogenous and one endogenous					
IS1	FESNFNTQATNR (52–63)	Lysozyme	19.5±2.0	714.8±2.4 (2+)	P00698
IS2	NTDGSTDYGILQINSR (64–79)	Lysozyme	36.2±2.0	877.4±2.4 (2+)	P00698
IS3	ITPNLAEFAFSLYR (50–63)	alpha-1-Antitrypsin	67.9±2.0	821.5±2.4 (2+)	P01009

a Residue numbers in the unprocessed precursor.

b Maximum range for all the analysis batches.

c Tolerance of ±0.5 *m/z* unit for the peak area calculation.

d (http://expasy.org/sprot/).

e *S*-Carboxyamidomethyl cysteine residue.

#### Signal normalization, signal alignment, and peak detection in the LC-MS data

Two-dimensional profile data consisting of LC retention time and full MS scan (LC-MS data) were extracted from all 220 LC-MS/MS measurements (Table 1) and processed using the i-OPAL algorithm (Patent # WO/2004/09526 AI).

i-OPAL is a dynamic programming algorithm that maximizes the alignment between LC-MS profiles through shrinking or extending the retention time axis to maximize the similarity in peak shapes within the chromatograms, and the similarity of the mass spectra, using a wider range of criteria than alternative data processing methods including dynamic time warping (DTW) [37] or correlation optimized warping (COW) [37], [38].

An important feature of the i-OPAL algorithm is its utilization of internal standards, which are forced to be aligned. This reduces the linear programming problem from the whole range of retention time to a series of small time sections, increasing the accuracy of alignment and reducing the computational time.

The i-OPAL program thus consists of 3 parts: 1) signal intensity normalization using one or more internal standards; 2) alignment of LC-MS data using internal standard signal sets and a dynamic programming algorithm; and 3) peak detection. First, the intensity of the whole signal was normalized across all samples using IS1. Second, alignment of the three internal standard signal sets (Table 3) was forced across the LC-MS data. Alignment of the remaining regions was carried out based on the dynamic programming algorithm. Following signal alignment, peak detection was performed using an iterative process.

A clear benefit of i-OPAL relative to other signal alignment approaches [39] is that peak detection is carried out after alignment rather than before. Most peak detection algorithms utilize the shape of peaks, making the integration of weak signals difficult. Aligning prior to peak detection increases the likelihood of detection of a peak across the range of samples, which can increase the numbers of peaks confidently detected.

### Statistical analysis and modeling of peptide data

First, Variance Scaling Normalization [40] was applied to the peaks to scale the signal intensities from each sample to a common level and also remove any dependency between the mean and variance of the intensities. PCA [41] of the scaled data was used for exploratory data analysis to identify the main sources of variation within the proteomic data.

The analysis to identify single peptide markers associated with case status then proceeded using ANCOVA, testing significance using type III sums of squares for each peptide separately, with normalized peptide intensity as the response and case/control status as the explanatory variable, with adjustment for some or all of the following covariates: analysis batch number and order within batch (both identified in the PCA), and the statistically significant clinical and radiological variables most predictive of ILD identified from the main study, i.e. WHO PS, smoking history, extent of normal lung coverage on CT scan and severity of pre-existing ILD [3]. Peptides were then ranked by the significance of the case-control status term.

To identify and evaluate the best set of peptide predictors, predictive multivariate modeling was then performed in two steps. First the *n* most significant peptides were selected and a PLS-DA [42] was performed to identify linear combinations of peptides correlating best with case-control status. This was followed by logistic regression modeling with case-control status as the response using the *m* first PLS components (linear combinations identified) as predictors. To explore the modeling space, the number of peptides, *n*, and the number of PLS components, *m*, utilized in the model were varied.

The performance of the statistical analysis and predictive multivariate modeling was assessed using a leave-one-out cross-validation approach to estimate sensitivity, specificity, and overall error rate of the modeling process [43], as follows:

- For each subject in turn, remove this subject as the test set, considering the remaining subjects as the training set.
- Perform the three steps of statistical analysis (ANCOVA and multivariate modeling by PLS-DA and logistic regression) as described above on the training set.
- Use the logistic regression model in the third step to predict the case-control status of the left-out subject based on the other data.
- Repeat, omitting each subject in turn from the training set and predicting the left-out subject and then combining the results to generate a vector of leave-one-out predictions, one for each subject, which can be used to give an estimate of the sensitivity and specificity as well as the overall error rate of the modeling process.

As a robustness check, Random Forests [44] were also used as an alternative to the combination of PLS-DA and logistic regression.

To allow a visualization of the potential choices for the appropriate levels of sensitivity and specificity, the cross-validated results are presented as ROC by varying the probability threshold used for predicting each subject as a case or a control.

### Peptide and protein identification

Product ion spectra with at least 10 product ions were converted into peak lists, which were searched with the Mascot algorithm [45] against a Swiss-Prot amino acid sequence database. First mass chromatogram files were generated for every LC-MS/MS measurement. The subsequent data conversion and search process was carried out using Mascot software (Version 2.1.04, Matrix Science Ltd, London, UK). Prior to database search, each product ion spectrum in these files was converted into peak list(s) using an extract_msn.exe program (Thermo Fisher Scientific) without any grouping process. The criterion for the data conversion was at least 10 product ions in a spectrum. These peak lists were searched with the Mascot algorithm (MS/MS Ion Search mode) [45] against a Swiss-Prot amino acid sequence database (http://www.expasy.org/sprot/; Release 55.0; 18,610 entries (*Homo sapiens*); updated on February 26, 2008). The database search parameters were set as follows: tryptic digestion (hydrolysis of the peptide bonds following lysine and arginine residues); fixed modification of cysteine residues (*S*-carboxyamidomethylation, +57.0 Da); variable modification of methionine residues (oxidation, +16.0 Da); ≤2 missed cleavages, i.e. assuming at most 2 predicted tryptic digestion sites are not actually digested; peptide tolerance of 2.0 Da; an MS/MS tolerance of ±0.8 Da (http://www.matrixscience.com).

### Bioinformatics

#### Identification of significant pathways

We utilized the IPA system (Ingenuity® Systems, www.ingenuity.com) to analyze the set of proteins we identified as demonstrating a significant difference in expression levels between cases and controls. The Ingenuity Pathways Analysis Knowledge Base is a large curated database of previously published findings on mammalian biology. The version used was v. 6.5, build 59570, content version 1602, build oqa-kb_enif, 2008-08-20, 21∶16∶03.

The list of proteins identified was overlaid onto the curated pathways in IPA. The dynamic Canonical Pathways are well-characterized metabolic and cell signaling pathways that have been curated and hand-drawn. The information contained in Canonical Pathways comes from specific journal articles, review articles, text books, and the KEGG (Kyoto Encyclopedia of Genes and Genomes) ligand database (http://www.genome.jp/kegg/ligand.html). The most significant pathway was identified as the pathway with the highest network score. The network score is based on the hypergeometric distribution and is calculated with the right-tailed Fisher's Exact Test. The score is the negative logarithm of this p-value. The ratio for an overlay to a given pathway was calculated by the ratio between the number of proteins from the data set found in the pathway and the total number of proteins associated to the pathway.

#### Gene network analysis

Gene network analysis was performed using IPA Systems web-based software application (http://www.ingenuity.com/products/pathways_analysis.html). In this approach the Ingenuity literature data are used to identify interconnected protein networks based on reported interactions identifying particular proteins as interacting with each other.

### Protein level modeling

For each of the proteins identified (through one or more peptides) as having the greatest significant difference between cases and controls, the full set of digested peptides from that protein was also identified, and a summary protein intensity (using the intensities of all component peptides) was calculated using a method based on PCA, as follows:

1. Remove the batch effect from all the peptide intensities by subtracting the batch means.
2. Restrict the set of peptides to those with a positive correlation after removing the batch effect with the peptide showing the greatest evidence of differential expression between the case and control groups (i.e. the smallest p-value).
3. Arrange the remaining batch adjusted peptide intensities in a matrix and apply PCA on the scaled data matrix.
4. The score of the first principal component, i.e. the greatest source of variability, is the protein intensity score.

Where there is strong correlation between all the constituent peptides, this method will produce effectively an average of the peptide intensities, as the loadings given to all the peptides will be similar. Where a peptide has low correlation with the remaining peptides, either because it is also a digestion product and therefore measuring other proteins, or has low intensity making it an unreliable measurement, then it will receive a low loading in the PCA and will contribute little to the protein intensity measure.

A summary pathway intensity score was calculated from the intensities of proteins within a common pathway in a similar manner by taking the scores from the first principal component of the matrix of protein intensities.

Protein and pathway intensities were modeled using logistic regression, with case-control status as the response and optionally the clinical variables as predictive variables, in an analogous way to peptide intensities as described above.

### Validation

Reproducibility of the identified differentially expressed peptides was validated by comparing the peptide intensities from 39 repeated samples with duplicate MS/MS analysis, allowing for consistent between-batch differences.

To validate between technologies, protein intensities derived from MS/MS were compared with those derived from Western blots with densitometry for 9 key selected proteins identified by the statistical and bioinformatic analyses. The depleted plasma proteins were reduced and denatured in the presence of 50 mM dithiothreitol and 2% w/v SDS at 95°C for 5 min, and then subjected to SDS-PAGE with 7.5%, 10%, or 12% acrylamide (0.1 or 1 µg protein *per* lane). Separated proteins were electrically transferred onto polyvinylidene difluoride (PVDF) membranes (Bio-Rad Laboratories) with a wet-type transfer system. After transfer, non-specific binding reactions were blocked by rocking the membranes for 1 h with 3% w/v BSA and 3% w/v polyvinylpyrrolidone K30 in TBST (150 mM NaCl, 0.05% v/v Tween 20 and 10 mM Tris-HCl, pH 8.0). The membranes were then washed with TBST, incubated with antibodies in TBST containing 0.1% BSA (TBST-BSA) for 30 min, washed again with TBST, and finally incubated with a HRP-conjugated second antibody in TBST-BSA for 30 min (Table S4). After washing again with TBST, the specific binding was detected using an ECL-Plus system (GE Healthcare) according to the manufacturer's instructions, in combination with a LAS-3000 imaging analyzer (FUJI FILM). For densitometry, the gel image was opened with Image-J software (National Institutes of Health, Research Services Branch, USA; http://rsbweb.nih.gov/), and the density of each band was obtained. After subtraction of a background value, the density data were used for further analyses.

## Supporting Information

Acknowledgment S1 **Members of the CCS study organization.**
(DOC)

Figure S1 **Comparison of p-values for all detected peptides from analyses adjusted for and unadjusted for batch.** All peptides showing a significant difference in the unadjusted analysis also show a significant difference in the analysis adjusted for batch. The analysis adjusted for batch also identifies additional significantly differentially expressed peptides that were not detected by the unadjusted analysis.
(TIF)

Figure S2 **Effect of batch-to-batch variability on the overall intensity difference between cases and controls for peptides.** (A) An example peptide which exhibits no discernable between-batch variation and so is significant independent of whether the analysis is adjusted for batch or not. (B) An example peptide with a highly significant variation between batches, which consequently is only significant when batch is accounted for. Overall pooled effect not adjusting for batch on left, followed by batches 1–9 with one study sample each from 180 of the 181 study subjects. Red  =  cases; blue  =  controls.
(TIF)

Figure S3 **Effect of adjusting for key clinical variables.** Left panel shows a comparison of p-values for all detected peptides from analyses adjusted for and unadjusted for 4 key clinical variables. Right panel shows an example of the effect of accounting for the clinical variable WHO PS on the pattern of intensity difference between cases and controls. Overall pooled effect on left, followed by case-control difference (red  =  cases; blue  =  controls) plotted by WHO PS.
(TIF)

Figure S4 **Networks obtained from entering the identified 29 proteins into the Ingenuity Pathway Analysis system.** Highest-scoring (A) and combined highest- and second highest-scoring (B) networks. Panel A represents a more annotated version of Figure 4 in the main manuscript material. In panel B, combining the two networks with the highest scores further adds IL1-beta, HNF1A, HNF4A, HNF6 (ONECUT1), and CEBPB as central components (green shading). In panel A, dark blue shapes and lines  =  proteins identified as predictors in this study and interactions between them. Grey shapes and lines  =  proteins identified by Ingenuity to generate the network and interactions between them. Light blue lines  =  interactions between proteins identified by Ingenuity to generate the network and the proteins identified in the study. **A** Relationship labels: A  =  Activation; B  =  Binding; C  =  Causes/Leads to; CC  =  Chemical-Chemical interaction; CP  =  Chemical-Protein interaction; E  =  Expression (includes metabolism/synthesis for chemicals); EC  =  Enzyme Catalysis; I  =  Inhibition; L  =  ProteoLysis (includes degradation for Chemicals); LO  =  Localization; M  =  Biochemical Modification; MB  =  Group/complex Membership; P  =  Phosphorylation/Dephosphorylation; PD  =  Protein-DNA binding; PP  =  Protein-Protein binding; PR  =  Protein-RNA binding; RB  =  Regulation of Binding; RE  =  Reaction; RR  =  RNA-RNA Binding; T  =  Transcription; TR  =  Translocation. Numbers in brackets  =  number of observations supporting the interaction. **B** Proteins identified in the study: SERPINA1  =  alpha-1-antitrypsin; SERPINA3  =  alpha-1-antichymotrypsin; SERPINC1  =  antithrombin-III; APOA1  =  apolipoprotein A-I; APOB  =  apolipoprotein B-100; APOC3  =  apolipoprotein C-III; C3  =  complement C3; C4A, C4B  =  complement C4-A; complement C4-B; C9  =  complement component C9; GSN  =  gelsolin; HBA2  =  hemoglobin alpha; HBB, HBD  =  hemoglobin beta/delta; HP  =  haptoglobin; HPR  =  haptoglobin-related protein; HRG  =  histidine-rich glycoprotein; KLKB1  =  plasma kallikrein; IGKC  =  Ig kappa chain V-III region Ti; RBP4, Rbp  =  retinol binding protein 4; APCS  =  serum amyloid P-component; TF  =  serotransferrin; TTR  =  transthyretin; ORM1  =  alpha-1-acid glycoprotein 1; A1BG  =  alpha-1B-glycoprotein; LRG1  =  leucine-rich alpha-2-glycoprotein; ARMC2  =  armadillo repeat-containing protein 2; AHSG  =  alpha-2-HS-glycoprotein; ITIH4  =  inter-alpha-trypsin inhibitor heavy chain H4.
(TIF)

Figure S5 **Scatterplots of intensities from Western blots (densitometry) and MS/MS for 9 selected differentially expressed proteins.** Red triangles  =  cases; green circles  =  controls. APOA1  =  apolipoprotein A–I; C3  =  complement C3; C4  =  complement C4-A; fetuin  =  alpha-2-HS-glycoprotein; HPT beta  =  haptoglobin; ORM1  =  alpha-1-acid glycoprotein; serpin A1  =  alpha-1-antitrypsin; serpin A3  =  alpha-1-antichymotrypsin.
(TIF)

Figure S6 **Western blot images from 12 subjects on 9 selected differentially expressed proteins.** 1 µg (0.1 µg for serpin A1) of depleted human plasma proteins of study samples (6 ILD cases and 6 controls) were separated by SDS-PAGE, transferred onto PVDF membrane, and detected with Western blotting. alpha-2-HS-glycoprotein  =  alpha-2-HS-glycoprotein; APOA1  =  apolipoprotein A-I; C3 beta-chain  =  complement C3 beta-chain; C4 beta-chain  =  complement C4 beta-chain; HPT beta-chain  =  haptoglobin beta-chain; orsomucoid-1  =  alpha-1-acid glycoprotein; serpin A1  =  alpha-1-antitrypsin; serpin A3  =  alpha-1-antichymotrypsin.
(TIF)

Figure S7 **Significance levels from proteins, constituent peptides, and acute phase pathway intensities, adjusted for clinical variables.** p-values for the proteins are shown by red stars, p-values for individual peptides are shown by points, and the distribution of these for each protein is shown by a boxplot. In each boxplot, the upper and lower sides of the box represent the higher and lower quartile values (Q3 and Q1), respectively. The black bar in each box represents the median value. The p-value for the acute phase pathway is represented by the dashed line; boxplots for proteins in the acute phase response pathway are shaded. A1AG1  =  alpha-1-acid glycoprotein; A1AT  =  alpha-1-antitrypsin; A1BG  =  alpha-1-B-glycoprotein; A2GL  =  leucine-rich alpha-2-glycoprotein; AACT  =  alpha-1-antichymotrypsin; ANT3  =  antithrombin-III; APOA1  =  apolipoprotein A–I; APOB  =  apolipoprotein B-100; APOC3  =  apolipoprotein C-III; ARMC2  =  armadillo repeat-containing protein 2; CO3  =  complement C3; CO4  =  complement C4-A, complement C4-B; CO9  =  complement component C9; FETUA  =  alpha-2-HS-glycoprotein; GELS  =  gelsolin; HBA  =  hemoglobin alpha; HBB,HBD  =  hemoglobin beta/delta; HPT  =  haptoglobin; HPTR  =  haptoglobin-related protein; HRG  =  histidine-rich glycoprotein; ITIH4  =  inter-alpha-trypsin inhibitor heavy chain H4; KLKB1  =  plasma kallikrein; KV3  =  Ig kappa chain V-III region Ti; RETBP  =  retinol binding protein 4; SAMP  =  serum amyloid P-component; TRFE  =  serotransferrin; TTHY  =  transthyretin.
(TIF)

Table S1 **Characteristics of study subjects (NSCLC patients treated with gefitinib) included in proteomics analyses.**
(DOC)

Table S2 **Quality control results of sample preparation for 181 study samples, by LC-MS/MS measurement batches.**
(DOC)

Table S3 **Protein Identification from the selected peaks.**
(DOC)

Table S4 **Antibodies used for Western Blot validation.**
(DOC)
