# Supplementary material for: Proteomic Biomarkers for Acute Interstitial Lung Disease in Gefitinib-Treated Japanese Lung Cancer Patients
Source: PLoS One. 2011 Jul 20;6(7):e22062. doi: 10.1371/journal.pone.0022062 (PMC3140475; doi:10.1371/journal.pone.0022062)
Supplement: Acknowledgment S1 — Members of the CCS study organization. (DOC) [file pone.0022062.s001.doc]

**Supporting Information**

## Acknowledgment S1

Members of the CCS study organization were as follows: **external Epidemiology Advisory Board**: Kenneth J Rothman, Jonathan M Samet, Toshiro Takezaki, Kotaro Ozasa, Masahiko Ando. **Case Review Board members** (including Japan Thoracic Radiology Group members*): Moritaka Suga, Takeshi Johkoh*, Masashi Takahashi*, Yoshiharu Ohno*, Sonoko Nagai, Yoshio Taguchi, Yoshikazu Inoue, Takashi Yana, Masahiko Kusumoto*, Hiroaki Arakawa*, Akinobu Yoshimura, Makoto Nishio, Yuichiro Ohe, Kunihiko Yoshimura, Hiroki Takahashi, Yukihiko Sugiyama, Masahito Ebina, Fumikazu Sakai*. **Hospitals and principal investigators**: National Hospital Organization Hokkaido Cancer Center (Hiroshi Isobe), Hokkaido University Hospital (Koichi Yamazaki), National Hospital Organization Dohoku National Hospital (Yuka Fujita), Tohoku University Hospital (Akira Inoue), Sendai Kousei Hospital (Shunichi Sugawara), National Cancer Center Hospital East (Yutaka Nishiwaki), Nippon Medical School Chiba Hokusoh Hospital (Yasushi Ono), Tokyo Medical University Hospital (Masahiro Tsuboi), Nippon Medical School Hospital (Tetsuya Okano), Toho University Omori Medical Center (Nobuyuki Hamanaka), Toranomon Hospital (Kunihiko Yoshimura), National Hospital Organization Tokyo Hospital (Atsuhisa Tamura), Juntendo University Hospital (Kazuhisa Takahashi), Kyorin University Hospital (Tomoyuki Goya), Tokai University Hospital (Kenji Eguchi), Kitasato University School of Medicine (Noriyuki Masuda), Kanagawa Cardiovascular and Respiratory Center (Takashi Ogura), Niigata Cancer Center Hospital (Akira Yokoyama), National Nishi-Niigata Central Hospital (Hiromi Miyao), Toyama University Hospital (Muneharu Maruyama), Kanazawa University Hospital (Kazuo Kasahara), Aichi Hospital, Aichi Cancer Center (Hiroshi Saito), National Hospital Organization Nagoya Medical Center (Hideo Saka), Fujita Health University Hospital (Hiroki Sakakibara), Nagoya Ekisaikai Hospital (Masashi Yamamoto), Shiga University of Medical Science Hospital (Noriaki Tezuka), Kyoto Katsura Hospital (Takeshi Hanawa), National Hospital Organization Kyoto Medical Center (Yoshiyuki Sasaki), Rinku General Medical Center Municipal Izumisano Hospital (Hisao Uejima), Kinki University, School of Medicine (Kazuhiko Nakagawa), National Hospital Organization Kinki-chuo Chest Medical Center (Masaaki Kawahara), Osaka City General Hospital (Koji Takeda), Osaka City General Hospital (Hirohito Tada), Osaka City University Hospital (Shinzoh Kudoh), Osaka Prefectural Medical Center for Respiratory and Allergic Diseases (Kaoru Matsui), Osaka Police Hospital (Kiyoshi Komuta), Toneyama National Hospital (Soichiro Yokota), Kobe City General Hospital (Keisuke Tomii), Hyogo Medical Center for Adults (Shunichi Negoro), Kobe University Hospital (Yoshihiro Nishimura), Institute of Biomedical Research and Innovation (Nobuyuki Katakami), Tenri Hospital (Yoshio Taguchi), Okayama University Medical and Dental School Hospital (Katsuyuki Kiura), Hiroshima City Hospital (Hidetaka Sumiyoshi), Hiroshima City Hospital (Noritomo Senoo), National Hospital Organization Shikoku Cancer Center (Tetsu Shinkai), National Hospital Organization Kyushu Cancer Center (Yukito Ichinose), Fukuoka National Hospital (Akira Motohiro), University of Occupational and Environmental Health (Masamitsu Kido), University of Occupational and Environmental Health (Kenji Sugio), National Hospital Organization Nagasaki Medical Center (Akitoshi Kinoshita), Kumamoto University Hospital (Mitsuhiro Matsumoto), Kumamoto-Chuo Hospital (Sunao Ushijima), Okinawa National Hospital (Mutsuo Kuba).
