# Supplementary figures and images for: Proteomic Biomarkers for Acute Interstitial Lung Disease in Gefitinib-Treated Japanese Lung Cancer Patients
Source: PLoS One. 2011 Jul 20;6(7):e22062. doi: 10.1371/journal.pone.0022062 (PMC3140475; doi:10.1371/journal.pone.0022062)

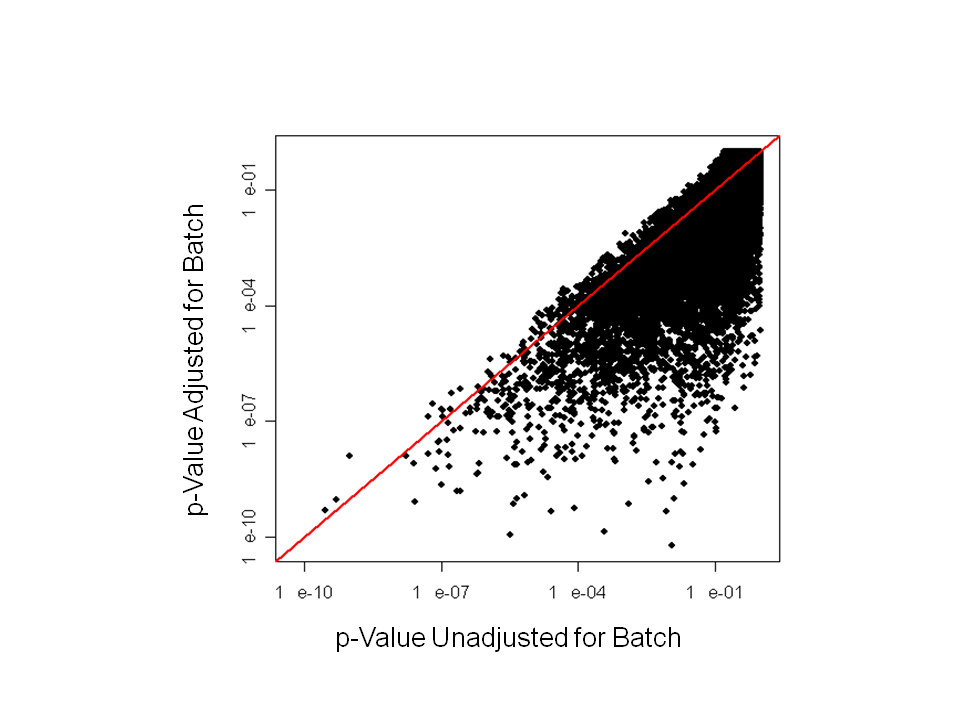

Supplement: Figure S1 — Comparison of p-values for all detected peptides from analyses adjusted for and unadjusted for batch. All peptides showing a significant difference in the unadjusted analysis also show a significant difference in the analysis adjusted for batch. The analysis adjusted for batch also identifies additional significantly differentially expressed peptides that were not detected by the unadjusted analysis. (TIF) [file pone.0022062.s002.tif]

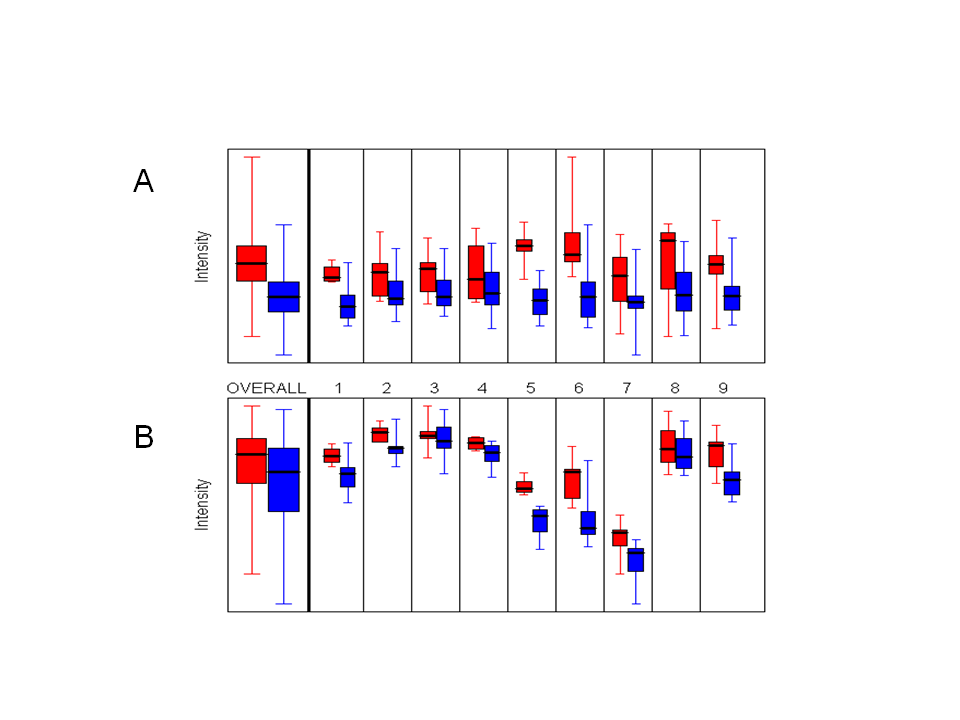

Supplement: Figure S2 — Effect of batch-to-batch variability on the overall intensity difference between cases and controls for peptides. (A) An example peptide which exhibits no discernable between-batch variation and so is significant independent of whether the analysis is adjusted for batch or not. (B) An example peptide with a highly significant variation between batches, which consequently is only significant when batch is accounted for. Overall pooled effect not adjusting for batch on left, followed by batches 1–9 with one study sample each from 180 of the 181 study subjects. Red = cases; blue = controls. (TIF) [file pone.0022062.s003.tif]

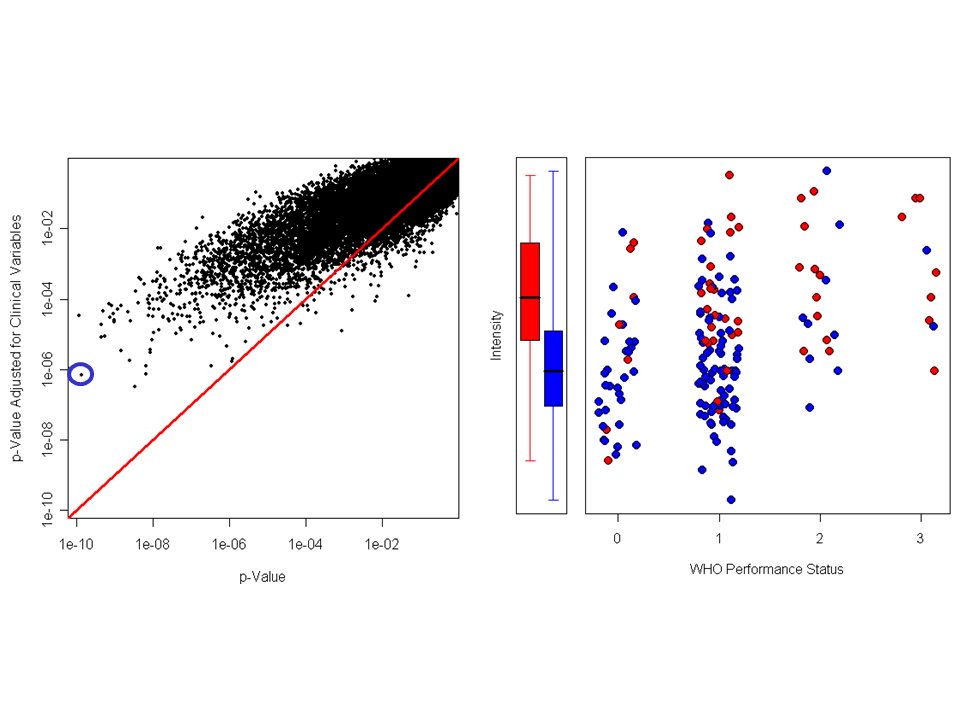

Supplement: Figure S3 — Effect of adjusting for key clinical variables. Left panel shows a comparison of p-values for all detected peptides from analyses adjusted for and unadjusted for 4 key clinical variables. Right panel shows an example of the effect of accounting for the clinical variable WHO PS on the pattern of intensity difference between cases and controls. Overall pooled effect on left, followed by case-control difference (red = cases; blue = controls) plotted by WHO PS. (TIF) [file pone.0022062.s004.tif]

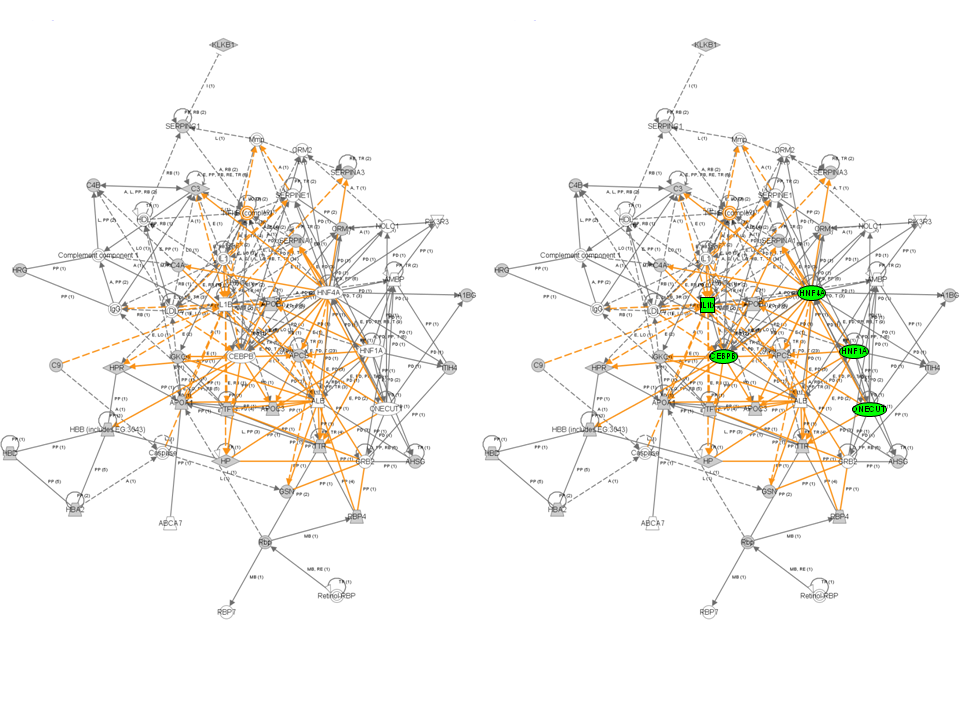

Supplement: Figure S4 — Networks obtained from entering the identified 29 proteins into the Ingenuity Pathway Analysis system. Highest-scoring (A) and combined highest- and second highest-scoring (B) networks. Panel A represents a more annotated version of Figure 4 in the main manuscript material. In panel B, combining the two networks with the highest scores further adds IL1-beta, HNF1A, HNF4A, HNF6 (ONECUT1), and CEBPB as central components (green shading). In panel A, dark blue shapes and lines = proteins identified as predictors in this study and interactions between them. Grey shapes and lines = proteins identified by Ingenuity to generate the network and interactions between them. Light blue lines = interactions between proteins identified by Ingenuity to generate the network and the proteins identified in the study. A Relationship labels: A = Activation; B = Binding; C = Causes/Leads to; CC = Chemical-Chemical interaction; CP = Chemical-Protein interaction; E = Expression (includes metabolism/synthesis for chemicals); EC = Enzyme Catalysis; I = Inhibition; L = ProteoLysis (includes degradation for Chemicals); LO = Localization; M = Biochemical Modification; MB = Group/complex Membership; P = Phosphorylation/Dephosphorylation; PD = Protein-DNA binding; PP = Protein-Protein binding; PR = Protein-RNA binding; RB = Regulation of Binding; RE = Reaction; RR = RNA-RNA Binding; T = Transcription; TR = Translocation. Numbers in brackets = number of observations supporting the interaction. B Proteins identified in the study: SERPINA1 = alpha-1-antitrypsin; SERPINA3 = alpha-1-antichymotrypsin; SERPINC1 = antithrombin-III; APOA1 = apolipoprotein A-I; APOB = apolipoprotein B-100; APOC3 = apolipoprotein C-III; C3 = complement C3; C4A, C4B = complement C4-A; complement C4-B; C9 = complement component C9; GSN = gelsolin; HBA2 = hemoglobin alpha; HBB, HBD = hemoglobin beta/delta; HP = haptoglobin; HPR = haptoglobin-related protein; HRG = histidine-rich glycoprotein; KLKB1 = plasma kallikrein; IGKC = [file pone.0022062.s005.tif]

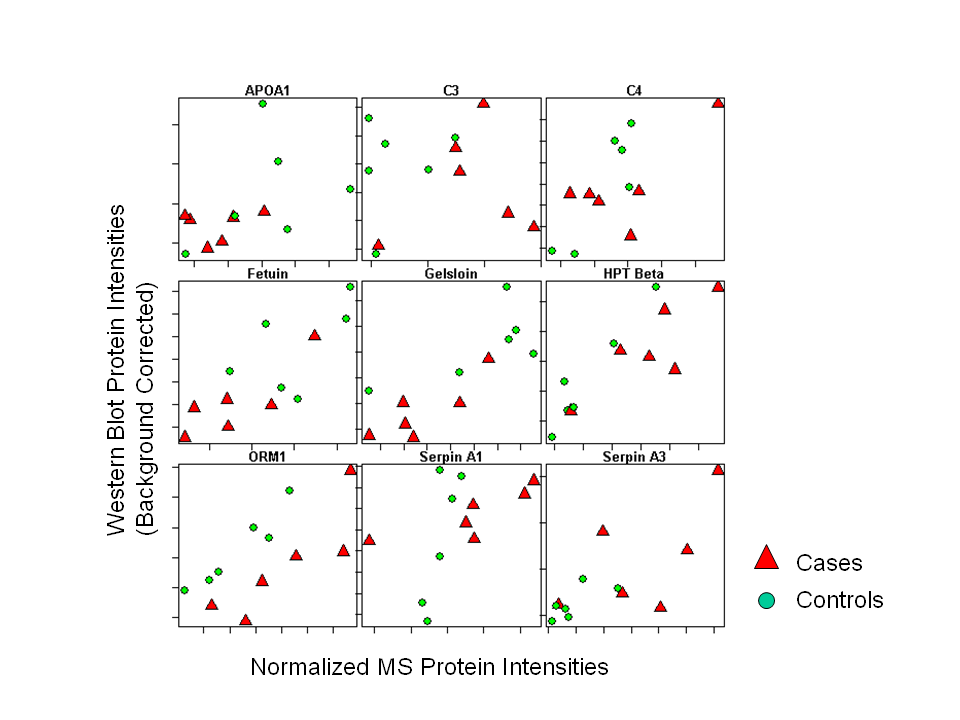

Supplement: Figure S5 — Scatterplots of intensities from Western blots (densitometry) and MS/MS for 9 selected differentially expressed proteins. Red triangles = cases; green circles = controls. APOA1 = apolipoprotein A–I; C3 = complement C3; C4 = complement C4-A; fetuin = alpha-2-HS-glycoprotein; HPT beta = haptoglobin; ORM1 = alpha-1-acid glycoprotein; serpin A1 = alpha-1-antitrypsin; serpin A3 = alpha-1-antichymotrypsin. (TIF) [file pone.0022062.s006.tif]

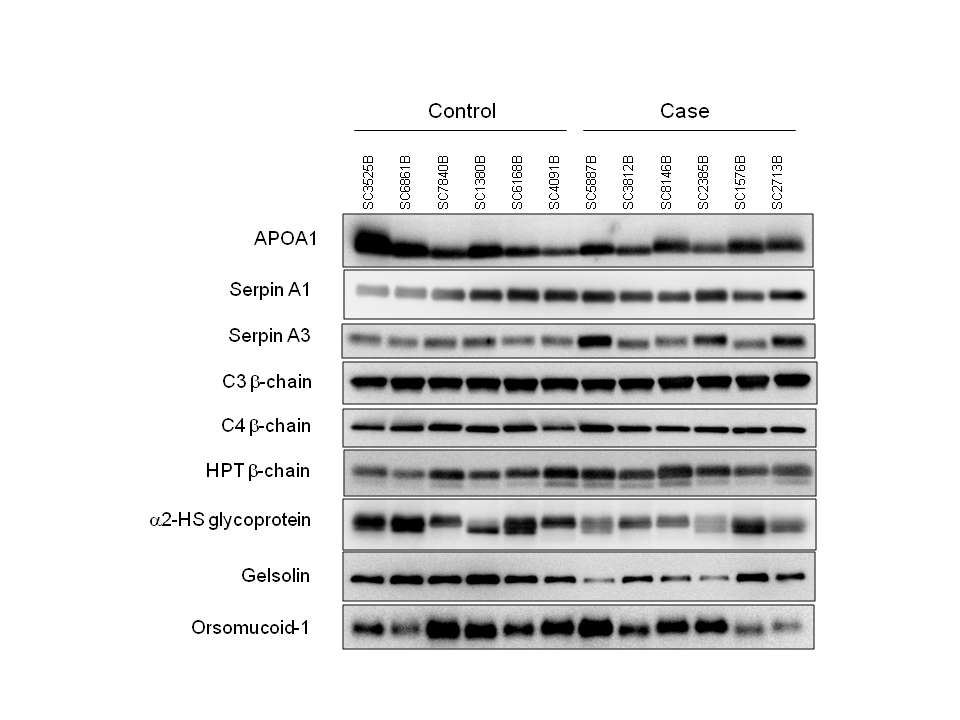

Supplement: Figure S6 — Western blot images from 12 subjects on 9 selected differentially expressed proteins. 1 µg (0.1 µg for serpin A1) of depleted human plasma proteins of study samples (6 ILD cases and 6 controls) were separated by SDS-PAGE, transferred onto PVDF membrane, and detected with Western blotting. alpha-2-HS-glycoprotein = alpha-2-HS-glycoprotein; APOA1 = apolipoprotein A-I; C3 beta-chain = complement C3 beta-chain; C4 beta-chain = complement C4 beta-chain; HPT beta-chain = haptoglobin beta-chain; orsomucoid-1 = alpha-1-acid glycoprotein; serpin A1 = alpha-1-antitrypsin; serpin A3 = alpha-1-antichymotrypsin. (TIF) [file pone.0022062.s007.tif]

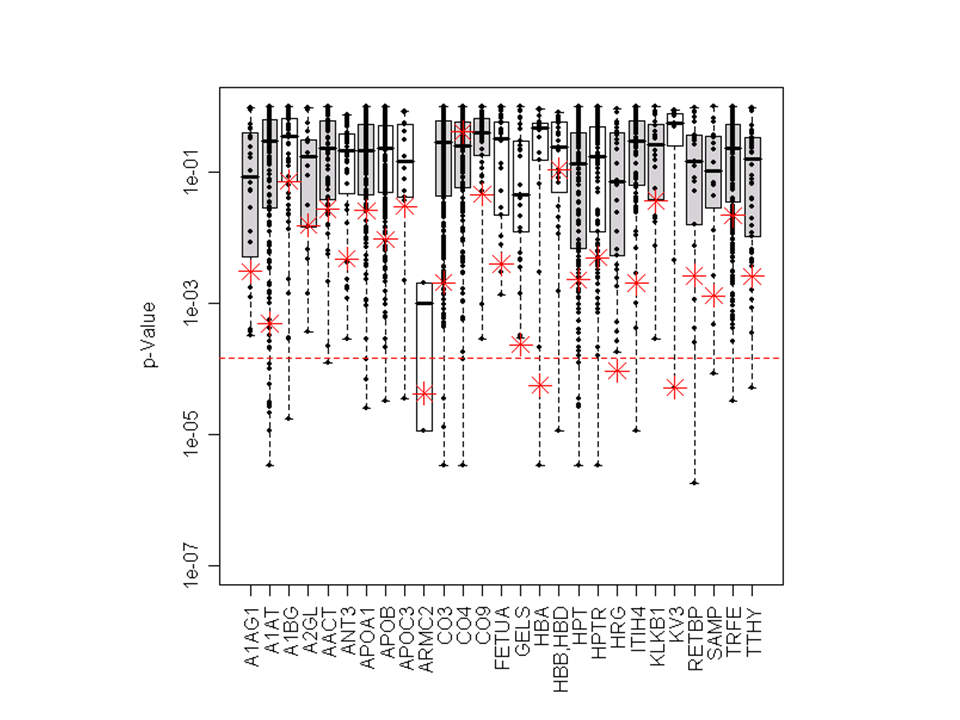

Supplement: Figure S7 — Significance levels from proteins, constituent peptides, and acute phase pathway intensities, adjusted for clinical variables. p-values for the proteins are shown by red stars, p-values for individual peptides are shown by points, and the distribution of these for each protein is shown by a boxplot. In each boxplot, the upper and lower sides of the box represent the higher and lower quartile values (Q3 and Q1), respectively. The black bar in each box represents the median value. The p-value for the acute phase pathway is represented by the dashed line; boxplots for proteins in the acute phase response pathway are shaded. A1AG1 = alpha-1-acid glycoprotein; A1AT = alpha-1-antitrypsin; A1BG = alpha-1-B-glycoprotein; A2GL = leucine-rich alpha-2-glycoprotein; AACT = alpha-1-antichymotrypsin; ANT3 = antithrombin-III; APOA1 = apolipoprotein A–I; APOB = apolipoprotein B-100; APOC3 = apolipoprotein C-III; ARMC2 = armadillo repeat-containing protein 2; CO3 = complement C3; CO4 = complement C4-A, complement C4-B; CO9 = complement component C9; FETUA = alpha-2-HS-glycoprotein; GELS = gelsolin; HBA = hemoglobin alpha; HBB,HBD = hemoglobin beta/delta; HPT = haptoglobin; HPTR = haptoglobin-related protein; HRG = histidine-rich glycoprotein; ITIH4 = inter-alpha-trypsin inhibitor heavy chain H4; KLKB1 = plasma kallikrein; KV3 = Ig kappa chain V-III region Ti; RETBP = retinol binding protein 4; SAMP = serum amyloid P-component; TRFE = serotransferrin; TTHY = transthyretin. (TIF) [file pone.0022062.s008.tif]
