# Supplementary material for: Proteomic Biomarkers for Acute Interstitial Lung Disease in Gefitinib-Treated Japanese Lung Cancer Patients
Source: PLoS One. 2011 Jul 20;6(7):e22062. doi: 10.1371/journal.pone.0022062 (PMC3140475; doi:10.1371/journal.pone.0022062)
Supplement: Table S1 — Characteristics of study subjects (NSCLC patients treated with gefitinib) included in proteomics analyses. (DOC) [file pone.0022062.s009.doc]

**Table S1.** Characteristics of Study Subjects (NSCLC Patients Treated with Gefitinib) Included in Proteomics Analyses.

|  | **Number (%)** | |
| --- | --- | --- |
|  | **ILD cases (n = 43)** | **Controls (n = 122)** |
| Sex  Male  Female | 28 (65.1) 15 (34.9) | 59 (48.3) 63 (51.6) |
| Age  <65 years  >65 years | 16 (37.2) 27 (62.3) | 57 (46.7) 65 (53.3) |
| Time since diagnosis of NSCLC  <0.5 years  0.5–<1 year  ≥1 year | 18 (41.2) 13 (30.2) 12 (27.9) | 33 (27.1) 33 (27.1) 56 (45.9) |
| WHO performance status  0  1  2–3 | 6 (13.4) 22 (51.2) 15 (34.9) | 30 (24.5) 80 (65.6) 12 (9.8) |
| Smoking history  No  Yes | 9 (21.4) 33 (78.6) | 53 (43.4) 69 (56.6) |
| Pre-existing interstitial lung disease on CT scan (CRB evaluation)  No ILD  Previous ILD  Unknown | 33 (76.7) 10 (23.3) 0 | 112 (94.1) 7 (5.8) 3 |
| Extent of normal lung on CT scan (CRB evaluation)  Low (10–50%)  Normal (60–100%)  Unknown | 24 (55.8) 19 (44.2) 0 | 24 (20.1) 95 (79.8) 3 |
