# Supplementary material for: Proteomic Biomarkers for Acute Interstitial Lung Disease in Gefitinib-Treated Japanese Lung Cancer Patients
Source: PLoS One. 2011 Jul 20;6(7):e22062. doi: 10.1371/journal.pone.0022062 (PMC3140475; doi:10.1371/journal.pone.0022062)
Supplement: Table S2 — Quality control results of sample preparation for 181 study samples, by LC-MS/MS measurement batches. (DOC) [file pone.0022062.s010.doc]

**Table S2.** Quality Control Results of Sample Preparation for 181 Study Samples, by LC-MS/MS Measurement Batches.

| **Position in LC-MS/MS batch^a^** |  | **Batch number** | | |  |  |  |  |  |  |  |  |  |  |  |  |  |  |  |  |  |
| --- | --- | --- | --- | --- | --- | --- | --- | --- | --- | --- | --- | --- | --- | --- | --- | --- | --- | --- | --- | --- | --- |
|  |  | **1** |  |  |  |  |  |  | **2** |  |  |  |  |  |  | **3** |  |  |  |  |  |
|  |  | **Patient ID^b^** | | **Depletion^c^** | |  | **Protein portion remaining after hydrolysis**  **(%)^d^** |  | **Patient ID** |  | **Depletion** |  |  | **Protein portion remaining after hydrolysis**  **(%)** |  | **Patient ID** |  | **Depletion** |  |  | **Protein portion remaining after hydrolysis**  **(%)** |
|  |  |  |  | **Batch number** | **Serum albumin in the depleted sample (%)** |  |  |  |  |  | **Batch number** | **Serum albumin in the depleted sample (%)** |  |  |  |  |  | **Batch number** | **Serum albumin in the depleted sample**  **(%)** |  |  |
|  |  |  |  |  |  |  |  |  |  |  |  |  |  |  |  |  |  |  |  |  |  |
| 2 |  | SC6908 |  | 6 | 3.1 |  | 18.0 |  | SC0448* |  | 7 | 3.7 |  | 16.0 |  | SC6959* |  | 15 | 6.1 |  | 16.4 |
| 3 |  | SC8591 |  | 9 | 3.2 |  | 23.0 |  | SC5479 |  | 13 | 4.4 |  | 16.0 |  | SC6156^#^ |  | 11 | 5.9 |  | 15.8 |
| 4 |  | SC4386 |  | 5 | 3.0 |  | 17.0 |  | SC1675 |  | 10 | 4.2 |  | 23.0 |  | SC8392^#^ |  | 9 | 4.6 |  | 4.0 |
| 5 |  | SC4146^#^ |  | 8 | 3.7 |  | 14.0 |  | SC9667 |  | 15 | 3.7 |  | 11.0 |  | SC8200 |  | 13 | 2.9 |  | 3.0 |
| 6 |  | SC7911^#^ |  | 14 | 4.4 |  | 18.0 |  | SC8009 |  | 6 | 3.6 |  | 11.0 |  | SC8444 |  | 6 | 3.5 |  | 9.9 |
| 7 |  | SC1285* |  | 12 | 3.3 |  | 14.0 |  | SC7209 |  | 13 | 4.1 |  | 17.0 |  | SC7256 |  | 13 | 4.3 |  | 11.7 |
| 8 |  | SC8628 |  | 14 | 3.7 |  | 19.0 |  | SC9742 |  | 15 | 5.1 |  | 20.0 |  | SC5203 |  | 5 | 3.1 |  | 10.5 |
| 9 |  | SC6120 |  | 13 | 4.7 |  | 10.0 |  | SC0719 |  | 16 | 5.7 |  | 23.0 |  | SC7905* |  | 15 | 4.0 |  | 18.0 |
| 10 |  | SC5911 |  | 8 | 2.8 |  | 13.0 |  | SC2908* |  | 15 | 3.5 |  | 18.0 |  | SC6429 |  | 15 | 4.4 |  | 19.0 |
| 11 |  | SC6669 |  | 2 | 4.7 |  | 20.0 |  | SC5058 |  | 5 | 3.5 |  | 16.0 |  | SC0979 |  | 1 | 3.8 |  | 15.0 |
| 13 |  | SC2310 |  | 14 | 3.2 |  | 11.0 |  | SC6124 |  | 9 | 2.6 |  | 16.0 |  | SC5581* |  | 8 | 4.0 |  | 23.7 |
| 14 |  | SC8387 |  | 14 | 4.5 |  | 23.0 |  | SC6322 |  | 11 | 2.3 |  | 20.3 |  | SC5681^#^ |  | 5 | 3.3 |  | 11.0 |
| 15 |  | SC7252* |  | 11 | 3.2 |  | 23.0 |  | SC6887^#^ |  | 6 | 2.9 |  | 8.0 |  | SC9607 |  | 13 | 3.3 |  | 13.3 |
| 16 |  | SC8271 |  | 13 | 4.1 |  | 24.0 |  | SC2929 |  | 7 | 2.7 |  | 19.0 |  | SC0821 |  | 12 | 3.6 |  | 6.7 |
| 17 |  | SC1615 |  | 14 | 4.0 |  | 18.0 |  | SC2083* |  | 1 | 4.4 |  | 20.0 |  | SC6877 |  | 15 | 3.8 |  | 11.9 |
| 18 |  | SC5921 |  | 13 | 5.1 |  | 17.0 |  | SC0968 |  | 15 | 3.8 |  | 20.0 |  | SC9618 |  | 6 | 3.3 |  | 7.5 |
| 19 |  | SC6030 |  | 5 | 3.5 |  | 17.0 |  | SC1621 |  | 3 | 4.2 |  | 19.0 |  | SC9901* |  | 15 | 7.9 |  | 13.3 |
| 20 |  | SC8597 |  | 11 | 3.4 |  | 12.2 |  | SC3476* |  | 12 | 3.1 |  | 20.0 |  | SC4734* |  | 5 | 2.6 |  | 14.5 |
| 21 |  | SC2459* |  | 14 | 3.8 |  | 17.0 |  | SC6228* |  | 15 | 3.5 |  | 21.0 |  | SC5429 |  | 11 | 4.7 |  | 11.5 |
| 22 |  | SC9011 |  | 6 | 3.3 |  | 19.0 |  | SC6811^#^ |  | 15 | 4.1 |  | 19.0 |  | SC0458 |  | 15 | 3.7 |  | 16.6 |

| **Position in LC-MS/MS batch^A^** |  | **Batch number** | | |  |  |  |  |  |  |  |  |  |  |  |  |  |  |  |  |  |
| --- | --- | --- | --- | --- | --- | --- | --- | --- | --- | --- | --- | --- | --- | --- | --- | --- | --- | --- | --- | --- | --- |
|  |  | **4** |  |  |  |  |  |  | **5** |  |  |  |  |  |  | **6** |  |  |  |  |  |
|  |  | **Patient ID^b^** | | **Depletion^c^** | |  | **Protein portion remaining after hydrolysis**  **(%)^d^** |  | **Patient ID** |  | **Depletion** |  |  | **Protein portion remaining after hydrolysis**  **(%)** |  | **Patient ID** |  | **Depletion** |  |  | **Protein portion remaining after hydrolysis**  **(%)** |
|  |  |  |  | **Batch number** | **Serum albumin in the depleted sample (%)** |  |  |  |  |  | **Batch number** | **Serum albumin in the depleted sample (%)** |  |  |  |  |  | **Batch number** | **Serum albumin in the depleted sample**  **(%)** |  |  |
|  |  |  |  |  |  |  |  |  |  |  |  |  |  |  |  |  |  |  |  |  |  |
| 2 |  | SC2645 |  | 2 | 2.7 |  | 15.0 |  | SC9266 |  | 13 | 3.6 |  | 18.0 |  | SC5887* |  | 16 | 3.9 |  | 16.8 |
| 3 |  | SC4045 |  | 10 | 3.2 |  | 14.7 |  | SC6419* |  | 5 | 3.1 |  | 20.3 |  | SC3812* |  | 16 | 3.4 |  | 23.4 |
| 4 |  | SC6533 |  | 15 | 3.9 |  | 12.7 |  | SC6527^#^ |  | 9 | 3.5 |  | 16.2 |  | SC9452 |  | 16 | 3.4 |  | 24.7 |
| 5 |  | SC5088* |  | 11 | 3.8 |  | 32.3 |  | SC9229* |  | 9 | 3.0 |  | 14.5 |  | SC6863 |  | 16 | 3.1 |  | 19.3 |
| 6 |  | SC3430 |  | 2 | 2.4 |  | 11.1 |  | SC1313 |  | 3 | 3.3 |  | 16.7 |  | SC1903 |  | 4 | 3.6 |  | 22.8 |
| 7 |  | SC6819* |  | 9 | 2.7 |  | 6.7 |  | SC3401* |  | 4 | 3.6 |  | 16.8 |  | SC2529^#^ |  | 10 | 6.4 |  | 22.5 |
| 8 |  | SC3344 |  | 12 | 4.3 |  | 15.1 |  | SC0544* |  | 15 | 4.5 |  | 17.6 |  | SC0074 |  | 1 | 3.3 |  | 17.3 |
| 9 |  | SC7286 |  | 13 | 2.3 |  | 12.1 |  | SC3704* |  | 7 | 3.8 |  | 18.3 |  | SC3525 |  | 4 | 2.4 |  | 22.8 |
| 10 |  | SC5918 |  | 13 | 5.0 |  | 13.6 |  | SC8727 |  | 11 | 3.0 |  | 15.7 |  | SC6861 |  | 6 | 2.9 |  | 19.8 |
| 11 |  | SC0393 |  | 15 | 3.9 |  | 8.0 |  | SC6999 |  | 6 | 3.9 |  | 16.1 |  | SC5015 |  | 10 | 3.2 |  | 20.4 |
| 13 |  | SC6357 |  | 5 | 2.8 |  | 24.6 |  | SC6784^#^ |  | 11 | 4.0 |  | 20.0 |  | SC6031 |  | 5 | 3.1 |  | 16.4 |
| 14 |  | SC0461 |  | 7 | 3.4 |  | 9.2 |  | SC0913 |  | 16 | 4.6 |  | 23.5 |  | SC8146* |  | 9 | 4.2 |  | 18.4 |
| 15 |  | SC1956 |  | 4 | 5.3 |  | 12.0 |  | SC3048 |  | 12 | 3.9 |  | 14.4 |  | SC6689 |  | 13 | 3.6 |  | 14.9 |
| 16 |  | SC4475^#^ |  | 15 | 3.1 |  | 14.3 |  | SC4831 |  | 18 | 3.2 |  | 9.6 |  | SC7840 |  | 13 | 2.6 |  | 15.5 |
| 17 |  | SC4114 |  | 15 | 4.5 |  | 10.8 |  | SC8797 |  | 13 | 2.5 |  | 16.4 |  | SC3034 |  | 12 | 3.5 |  | 21.0 |
| 18 |  | SC4381 |  | 4 | 3.2 |  | 15.5 |  | SC4586* |  | 16 | 2.9 |  | 17.4 |  | SC4223 |  | 12 | 4.8 |  | 13.2 |
| 19 |  | SC2455* |  | 12 | 3.6 |  | 19.4 |  | SC4923 |  | 16 | 5.8 |  | 13.8 |  | SC5533 |  | 5 | 3.2 |  | 21.9 |
| 20 |  | SC9480 |  | 6 | 2.8 |  | 14.3 |  | SC7707 |  | 16 | 4.0 |  | 11.7 |  | SC4488 |  | 16 | 4.5 |  | 16.5 |
| 21 |  | SC4330 |  | 15 | 3.9 |  | 13.5 |  | SC0423 |  | 1 | 4.4 |  | 16.7 |  | SC0709* |  | 1 | 6.5 |  | 15.1 |
| 22 |  | SC0872^#^ |  | 3 | 3.1 |  | 15.1 |  | SC2176 |  | 16 | 4.0 |  | 17.9 |  | SC8475* |  | 9 | 2.8 |  | 20.3 |

| **Position in LC-MS/MS batch^a^** |  | **Batch number** | | |  |  |  |  |  |  |  |  |  |  |  |  |  |  |  |  |  |
| --- | --- | --- | --- | --- | --- | --- | --- | --- | --- | --- | --- | --- | --- | --- | --- | --- | --- | --- | --- | --- | --- |
|  |  | **7** |  |  |  |  |  |  | **8** |  |  |  |  |  |  | **9** |  |  |  |  |  |
|  |  | **Patient ID^b^** | | **Depletion^c^** | |  | **Protein portion remaining after hydrolysis**  **(%)^d^** |  | **Patient ID** |  | **Depletion** |  |  | **Protein portion remaining after hydrolysis**  **(%)** |  | **Patient ID** |  | **Depletion** |  |  | **Protein portion remaining after hydrolysis**  **(%)** |
|  |  |  |  | **Batch number** | **Serum albumin in the depleted sample (%)** |  |  |  |  |  | **Batch number** | **Serum albumin in the depleted sample (%)** |  |  |  |  |  | **Batch number** | **Serum albumin in the depleted sample**  **(%)** |  |  |
|  |  |  |  |  |  |  |  |  |  |  |  |  |  |  |  |  |  |  |  |  |  |
| 2 |  | SC1380 |  | 16 | 3.9 |  | 7.8 |  | SC1640* |  | 7 | 3.6 |  | 18.4 |  | SC5664^#^ |  | 11 | 3.7 |  | 12.0 |
| 3 |  | SC6168 |  | 17 | 4.6 |  | 10.3 |  | SC6412* |  | 17 | 3.6 |  | 15.4 |  | SC5077* |  | 17 | 3.1 |  | 6.0 |
| 4 |  | SC4091 |  | 17 | 2.8 |  | 12.3 |  | SC5122 |  | 8 | 3.0 |  | 13.4 |  | SC7237 |  | 17 | 3.4 |  | 10.0 |
| 5 |  | SC3839 |  | 12 | 3.4 |  | 6.8 |  | SC0756 |  | 3 | 3.3 |  | 17.9 |  | SC6053* |  | 5 | 3.0 |  | 16.0 |
| 6 |  | SC2385* |  | 17 | 4.1 |  | 11.9 |  | SC7371 |  | 17 | 3.9 |  | 15.5 |  | SC5719* |  | 13 | 3.1 |  | 16.0 |
| 7 |  | SC4963 |  | 12 | 3.6 |  | 13.8 |  | SC7632 |  | 2 | 4.1 |  | 14.8 |  | SC0591* |  | 1 | 3.8 |  | 14.0 |
| 8 |  | SC3402 |  | 12 | 3.6 |  | 13.1 |  | SC9214 |  | 17 | 2.2 |  | 15.8 |  | SC4326 |  | 4 | 4.0 |  | 14.0 |
| 9 |  | SC1576* |  | 10 | 4.7 |  | 10.8 |  | SC3718 |  | 12 | 2.7 |  | 17.8 |  | SC9542 |  | 17 | 3.4 |  | 20.0 |
| 10 |  | SC6214 |  | 13 | 4.2 |  | 11.1 |  | SC1652 |  | 3 | 4.6 |  | 16.7 |  | SC8405 |  | 17 | 3.1 |  | 10.8 |
| 11 |  | SC4924 |  | 17 | 4.4 |  | 11.7 |  | SC5977 |  | 13 | 4.3 |  | 14.2 |  | SC3110 |  | 12 | 4.7 |  | 12.0 |
| 13 |  | SC9099^#^ |  | 17 | 4.2 |  | 10.8 |  | SC4123 |  | 4 | 3.4 |  | 14.0 |  | SC0459 |  | 17 | 3.5 |  | 11.3 |
| 14 |  | SC9838 |  | 16 | 4.7 |  | 10.0 |  | SC4332* |  | 17 | 3.0 |  | 16.8 |  | SC7817 |  | 6 | 4.3 |  | 14.0 |
| 15 |  | SC8452 |  | 6 | 3.3 |  | 12.2 |  | SC3182 |  | 4 | 3.9 |  | 17.5 |  | SC8901 |  | 11 | 3.9 |  | 10.5 |
| 16 |  | SC2713* |  | 4 | 4.0 |  | 14.3 |  | SC5190 |  | 12 | 4.1 |  | 12.0 |  | SC7139 |  | 13 | 3.5 |  | 9.4 |
| 17 |  | SC5214* |  | 8 | 2.7 |  | 11.0 |  | SC2880^#^ |  | 10 | 4.2 |  | 13.1 |  | SC0725 |  | 7 | 3.2 |  | 10.1 |
| 18 |  | SC1372 |  | 3 | 4.0 |  | 18.0 |  | SC2576 |  | 12 | 4.0 |  | 15.6 |  | SC8033* |  | 2 | 3.4 |  | 7.4 |
| 19 |  | SC2810* |  | 4 | 3.9 |  | 16.9 |  | SC7818 |  | 6 | 2.1 |  | 5.6 |  | SC7080^#^ |  | 9 | 4.1 |  | 12.7 |
| 20 |  | SC6403 |  | 5 | 2.8 |  | 11.7 |  | SC0144 |  | 17 | 3.6 |  | 15.0 |  | SC3418 |  | 4 | 3.8 |  | 15.3 |
| 21 |  | SC6123* |  | 2 | 3.0 |  | 13.4 |  | SC3459 |  | 2 | 2.8 |  | 15.0 |  | SC3155 |  | 12 | 5.5 |  | 18.3 |
| 22 |  | SC5298 |  | 8 | 3.4 |  | 14.1 |  | SC3929 |  | 17 | 4.7 |  | 21.7 |  | SC4214* |  | 4 | 3.7 |  | 13.5 |

| **Position in LC-MS/MS batch^a^** |  | **Batch number** | | |  |  |  |  |  |  |  |  |  |  |  |  |  |  |  |  |  |
| --- | --- | --- | --- | --- | --- | --- | --- | --- | --- | --- | --- | --- | --- | --- | --- | --- | --- | --- | --- | --- | --- |
|  |  | **10^e^** |  |  |  |  |  |  | **11^f^** |  |  |  |  |  |  |  |  |  |  |  |  |
|  |  | **Patient ID^b^** | | **Depletion^c^** | |  | **Protein portion remaining after hydrolysis**  **(%)^d^** |  | **Patient ID** |  | **Depletion** |  |  | **Protein portion remaining after hydrolysis**  **(%)** |  |  |  |  |  |  |  |
|  |  |  |  | **Batch number** | **Serum albumin in the depleted sample (%)** |  |  |  |  |  | **Batch number** | **Serum albumin in the depleted sample (%)** |  |  |  |  |  |  |  |  |  |
|  |  |  |  |  |  |  |  |  |  |  |  |  |  |  |  |  |  |  |  |  |  |
| 2 |  | SC6908 |  | 6 | 3.1 |  | 18.0 |  | SC6959* |  | 15 | 6.1 |  | 16.4 |  |  |  |  |  |  |  |
| 3 |  | SC8591 |  | 9 | 3.2 |  | 23.0 |  | SC6156^#^ |  | 11 | 5.9 |  | 15.8 |  |  |  |  |  |  |  |
| 4 |  | SC4386 |  | 5 | 3.0 |  | 17.0 |  | *SC8392*^#^ |  | 9 | 4.6 |  | 4.0 |  |  |  |  |  |  |  |
| 5 |  | SC4146^#^ |  | 8 | 3.7 |  | 14.0 |  | SC8200 |  | 13 | 2.9 |  | 3.0 |  |  |  |  |  |  |  |
| 6 |  | SC7911^#^ |  | 14 | 4.4 |  | 18.0 |  | SC8444 |  | 6 | 3.5 |  | 9.9 |  |  |  |  |  |  |  |
| 7 |  | SC1285* |  | 12 | 3.3 |  | 14.0 |  | SC7256 |  | 13 | 4.3 |  | 11.7 |  |  |  |  |  |  |  |
| 8 |  | SC8628 |  | 14 | 3.7 |  | 19.0 |  | SC5203 |  | 5 | 3.1 |  | 10.5 |  |  |  |  |  |  |  |
| 9 |  | SC6120 |  | 13 | 4.7 |  | 10.0 |  | SC7905* |  | 15 | 4.0 |  | 18.0 |  |  |  |  |  |  |  |
| 10 |  | SC5911 |  | 8 | 2.8 |  | 13.0 |  | SC6429 |  | 15 | 4.4 |  | 19.0 |  |  |  |  |  |  |  |
| 11 |  | SC6669 |  | 2 | 4.7 |  | 20.0 |  | SC0979 |  | 1 | 3.8 |  | 15.0 |  |  |  |  |  |  |  |
| 13 |  | SC2310 |  | 14 | 3.2 |  | 11.0 |  | SC5581* |  | 8 | 4.0 |  | 23.7 |  |  |  |  |  |  |  |
| 14 |  | SC8387 |  | 14 | 4.5 |  | 23.0 |  | SC5681^#^ |  | 5 | 3.3 |  | 11.0 |  |  |  |  |  |  |  |
| 15 |  | SC7252* |  | 11 | 3.2 |  | 23.0 |  | SC9607 |  | 13 | 3.3 |  | 13.3 |  |  |  |  |  |  |  |
| 16 |  | SC8271 |  | 13 | 4.1 |  | 24.0 |  | SC0821 |  | 12 | 3.6 |  | 6.7 |  |  |  |  |  |  |  |
| 17 |  | SC1615 |  | 14 | 4.0 |  | 18.0 |  | SC6877 |  | 15 | 3.8 |  | 11.9 |  |  |  |  |  |  |  |
| 18 |  | SC4729 |  | 12 | 2.8 |  | 13.6 |  | SC9618 |  | 6 | 3.3 |  | 7.5 |  |  |  |  |  |  |  |
| 19 |  | SC6030 |  | 5 | 3.5 |  | 17.0 |  | SC9901* |  | 15 | 7.9 |  | 13.3 |  |  |  |  |  |  |  |
| 20 |  | SC8597 |  | 11 | 3.4 |  | 12.2 |  | SC4734* |  | 5 | 2.6 |  | 14.5 |  |  |  |  |  |  |  |
| 21 |  | SC2459* |  | 14 | 3.8 |  | 17.0 |  | SC5429 |  | 11 | 4.7 |  | 11.5 |  |  |  |  |  |  |  |
| 22 |  | SC9011 |  | 6 | 3.3 |  | 19.0 |  | SC0458 |  | 15 | 3.7 |  | 16.6 |  |  |  |  |  |  |  |

^a^Experimental control samples were analyzed at the 1st, 12th, and 23rd positions.

^b^In batches 1–9, ILD cases confirmed by the Case Review Board (n = 42) are indicated with an asterisk (*). Clinically diagnosed cases rejected by the Case Review Board (n = 15) are shown with ^#^. One rejected control (SC8392), rejected because later developed ILD and included as confirmed case (with another sample number), is indicated in italic with ^#^. Repeated samples in batches 10–11 are similarly marked.

^c^The 181 distinct samples were depleted in 18 depletion batches and randomized across and within 11 LC-MS/MS batches.

^d^The 181 distinct samples were included in a single hydrolysis batch.

^e^Repeated analysis of 19 samples from batch 1. SC4729 was the only distinct sample in this batch.

^f^Repeated analysis of all 20 samples in batch 3.
