# Supplementary material for: Proteomic Biomarkers for Acute Interstitial Lung Disease in Gefitinib-Treated Japanese Lung Cancer Patients
Source: PLoS One. 2011 Jul 20;6(7):e22062. doi: 10.1371/journal.pone.0022062 (PMC3140475; doi:10.1371/journal.pone.0022062)
Supplement: Table S3 — Protein Identification from the selected peaks. (DOC) [file pone.0022062.s011.doc]

**Table S3.** Protein Identification from the Selected Peaks.^a^

| **Protein name** | **Uniprot** | **Molecular** | **Peak** |  | **Amino acid sequence of peptide** | **Charge** | **Normalized** | **Normalized** | **p-value** |  | **Maximum** |  |  | **Number of samples** | | |
| --- | --- | --- | --- | --- | --- | --- | --- | --- | --- | --- | --- | --- | --- | --- | --- | --- |
|  | **accession** | **weight** | **number** |  |  | **state** | **retention time** | ***m/z*** |  |  | **Mascot score** |  | **ILD**  **case** | **Control** | **RC^c^** | **Total** |
| alpha-1-acid glycoprotein 1 | P02763 | 23,512 | 20 |  | YVGGQEHFAHLLILR | 3 | 38.1 | 585.7 | 4.41E-09 |  | 75 |  | 37 | 109 | 15 | 161 |
|  |  |  | 19 |  | YVGGQEHFAHLLILR | 2 | 38.0 | 877.8 | 2.04E-08 |  | 88 |  | 46 | 130 | 16 | 192 |
|  |  |  | 15^b^ |  | YVGGQEHFAHLLILR | 3 | 40.3 | 583.9 | 3.82E-08 |  | 49 |  | 4 | 3 | 0 | 7 |
|  |  |  | 21 |  | YVGGQEHFAHLLILR | 3 | 41.2 | 586.9 | 1.66E-03 |  | 53 |  | 2 | 1 | 0 | 3 |
| alpha-1-antitrypsin | P01009 | 46,737 | 8^b^ |  | VFSNGADLSGVTEEAPLK | 2 | 36.9 | 918.4 | 1.51E-09 |  | 116 |  | 46 | 137 | 17 | 200 |
|  |  |  | 11 |  | ITPNLAEFAFSLYR | 3 | 66.4 | 548.5 | 7.46E-09 |  | 87 |  | 45 | 136 | 17 | 198 |
|  |  |  | 10 |  | LSSWVLLMK | 2 | 49.1 | 539.4 | 1.02E-08 |  | 69 |  | 42 | 117 | 17 | 176 |
|  |  |  | 18^b^ |  | KLSSWVLLMK | 2 | 43.2 | 603.3 | 1.05E-08 |  | 64 |  | 46 | 129 | 17 | 192 |
|  |  |  | 7^b^ |  | LYHSEAFTVNFGDTEEAKK | 3 | 33.2 | 730.2 | 2.53E-08 |  | 100 |  | 45 | 133 | 15 | 193 |
|  |  |  | 5 |  | RLGMFNIQHCK | 3 | 23.6 | 469.0 | 6.94E-08 |  | 64 |  | 27 | 71 | 12 | 110 |
|  |  |  | 12 |  | ITPNLAEFAFSLYR | 2 | 66.4 | 821.8 | 7.11E-08 |  | 99 |  | 46 | 137 | 17 | 200 |
|  |  |  | 6 |  | KLYHSEAFTVNFGDTEEAKK | 3 | 29.9 | 772.7 | 1.75E-07 |  | 77 |  | 2 | 11 | 3 | 16 |
|  |  |  | 9 |  | VFSNGADLSGVTEEAPLKLSK | 3 | 39.8 | 722.0 | 1.52E-06 |  | 92 |  | 34 | 97 | 15 | 146 |
| alpha-1B-glycoprotein | P04217 | 54,273 | 38 |  | TPGAAANLELIFVGPQHAGNYR | 2 | 48.0 | 1,148.5 | 8.13E-06 |  | 94 |  | 0 | 5 | 0 | 5 |
| Leucine-rich alpha-2-glycoprotein | P02750 | 38,178 | 31 |  | DLLLPQPDLR | 2 | 41.1 | 590.0 | 4.73E-09 |  | 64 |  | 21 | 21 | 3 | 45 |
| alpha-1-antichymotrypsin | P01011 | 47,651 | 25 |  | EQLSLLDRFTEDAKR | 3 | 39.9 | 608.7 | 8.30E-09 |  | 72 |  | 31 | 90 | 11 | 132 |
| Antithrombin-III | P01008 | 52,602 | 16 |  | AFLEVNEEGSEAAASTAVVIAGR | 2 | 47.1 | 1,146.8 | 5.85E-08 |  | 99 |  | 8 | 50 | 5 | 63 |
| Apolipoprotein A-I | P02647 | 30,778 | 2 |  | LREQLGPVTQEFWDNLEK | 2 | 52.5 | 1,102.8 | 1.51E-07 |  | 106 |  | 34 | 120 | 14 | 168 |
| Apolipoprotein B-100 | P04114 | 515,563 | 23^b^ |  | NHLQLEGLFFTNGEHTSK | 3 | 42.7 | 691.8 | 7.77E-08 |  | 77 |  | 15 | 55 | 5 | 75 |
| Apolipoprotein C-III | P02656 | 10,852 | 28^b^ |  | GWVTDGFSSLK | 2 | 39.6 | 599.3 | 1.03E-06 |  | 72 |  | 5 | 6 | 0 | 11 |
| Armadillo repeat-containing protein 2 | Q8NEN0 | 96,867 | 18^b^ |  | KLYIAELLLK | 2 | 43.2 | 603.3 | 1.05E-08 |  | 57 |  | 21 | 29 | 6 | 56 |
| Complement C3 | P01024 | 187,148 | 8^b^ |  | EGVQKEDIPPADLSDQVPDTESETR | 3 | 36.9 | 918.4 | 1.51E-09 |  | 80 |  | 14 | 65 | 7 | 86 |
|  |  |  | 3 |  | TELRPGETLNVNFLLR | 2 | 46.9 | 937.9 | 5.44E-08 |  | 72 |  | 12 | 34 | 3 | 49 |
|  |  |  | 28^b^ |  | VELLHNPAFCSLATTK | 3 | 39.6 | 599.3 | 1.03E-06 |  | 64 |  | 4 | 3 | 1 | 8 |
| Complement C4-A, complement C4-B | P0C0L4,P0C0L5 | 192,771, 192,793 | 8^b^ |  | VQQPDCREPFLSCCQFAESLRK | 3 | 36.9 | 918.4 | 1.51E-09 |  | 61 |  | 1 | 11 | 1 | 13 |
|  |  |  | 30 |  | AVGSGATFSHYYYMILSR | 2 | 49.9 | 1,011.8 | 5.30E-08 |  | 62 |  | 0 | 4 | 0 | 4 |
| Complement component C9 | P02748 | 63,173 | 35^b^ |  | CLCACPFKFEGIACEISK | 3 | 45.9 | 733.3 | 6.73E-08 |  | 59 |  | 8 | 10 | 2 | 20 |
| Plasma kallikrein | P03952 | 71,370 | 35^b^ |  | CQFFTYSLLPEDCKEEK | 3 | 45.9 | 733.3 | 6.73E-08 |  | 55 |  | 4 | 10 | 1 | 15 |
| alpha-2-HS-glycoprotein | P02765 | 39,325 | 26 |  | TVVQPSVGAAAGPVVPPCPGR | 2 | 30.2 | 1,009.2 | 2.86E-08 |  | 107 |  | 38 | 132 | 14 | 184 |
| Gelsolin | P06396 | 85,698 | 4 |  | NWRDPDQTDGLGLSYLSSHIANVER | 3 | 48.9 | 949.6 | 9.88E-09 |  | 106 |  | 23 | 110 | 10 | 143 |
| Hemoglobin alpha | P69905 | 15,258 | 8^b^ |  | TYFPHFDLSHGSAQVK | 2 | 36.9 | 918.4 | 1.51E-09 |  | 75 |  | 1 | 2 | 0 | 3 |
| Hemoglobin beta/delta | P68871/P02042 | 15,998/ 16,055 | 18^b^ |  | KVLGAFSDGLAHLDNLK | 3 | 43.2 | 603.3 | 1.05E-08 |  | 66 |  | 1 | 3 | 0 | 4 |
| Haptoglobin | P00738 | 45,205 | 8^b^ |  | VMPICLPSKDYAEVGR | 2 | 36.9 | 918.4 | 1.51E-09 |  | 75 |  | 24 | 75 | 8 | 107 |
|  |  |  | 7^b^ |  | SPVGVQPILNEHTFCAGMoxSK | 3 | 33.2 | 730.2 | 2.53E-08 |  | 58 |  | 20 | 54 | 8 | 82 |
|  |  |  | 28^b^ |  | VVLHPNYSQVDIGLIK | 3 | 39.6 | 599.3 | 1.03E-06 |  | 78 |  | 39 | 78 | 15 | 132 |
| Haptoglobin-related protein | P00739 | 39,008 | 8^b^ |  | VMPICLPSKNYAEVGR | 2 | 36.9 | 918.4 | 1.51E-09 |  | 77 |  | 32 | 83 | 14 | 129 |
| Histidine-rich glycoprotein | P04196 | 59,578 | 15^b^ |  | VRGGEGTGYFVDFSVR | 3 | 40.3 | 583.9 | 3.82E-08 |  | 62 |  | 5 | 22 | 1 | 28 |
| Inter-alpha-trypsin inhibitor heavy chain H4 | Q14624 | 103,325 | 18^b^ |  | RLGVYELLLK | 2 | 43.2 | 603.3 | 1.05E-08 |  | 54 |  | 5 | 5 | 1 | 11 |
| Retinol binding protein 4 | P02753 | 23,010 | 29 |  | GNDDHWIVDTDYDTYAVQYSCR | 3 | 45.3 | 898.1 | 2.43E-09 |  | 49 |  | 2 | 6 | 3 | 11 |
| Serum amyloid P-component | P02743 | 25,387 | 27 |  | AYSLFSYNTQGRDNELLVYK | 3 | 46.9 | 795.2 | 1.73E-08 |  | 56 |  | 19 | 38 | 5 | 62 |
| Serotransferrin | P02787 | 77,050 | 23^b^ |  | SDNCEDTPEAGYFAVAVVK | 3 | 42.7 | 691.8 | 7.77E-08 |  | 67 |  | 4 | 21 | 5 | 30 |
| Transthyretin | P02766 | 15,887 | 36^b^ |  | ALGISPFHEHAEVVFTANDSGPR | 3 | 37.2 | 818.2 | 1.07E-08 |  | 84 |  | 26 | 91 | 9 | 126 |
| Ig kappa chain V-III region Ti | P01622 | 11,788 | 36^b^ |  | FSGSGSGTDFTLTISR | 2 | 37.2 | 818.2 | 1.07E-08 |  | 91 |  | 25 | 74 | 12 | 111 |

^a^Of the 41 selected peptide peaks, 28 contain the total 45 peptide identifications including two peptides from the spiked lysozyme (peak nos 18 and 19). The other 13 peaks contain no peptide identification meeting the provided criteria. This table shows the plasma-derived 43 peptide identifications, composing 27 protein identifications, of which 2 are dual identifications to 2 closely related proteins in the same family (complement C4-A/complement C4-B and hemoglobin beta/delta).

^b^These peaks contain at least 2 peptide identifications.

^c^RC = Rejected Case (clinically diagnosed ILD cases where diagnosis was rejected by Case Review Board on blinded review of diagnostic data.
