# Supplementary material for: Proteomic Biomarkers for Acute Interstitial Lung Disease in Gefitinib-Treated Japanese Lung Cancer Patients
Source: PLoS One. 2011 Jul 20;6(7):e22062. doi: 10.1371/journal.pone.0022062 (PMC3140475; doi:10.1371/journal.pone.0022062)
Supplement: Table S4 — Antibodies used for Western Blot validation. (DOC) [file pone.0022062.s012.doc]

**Table S4.** Antibodies Used for Western Blot Validation.

| **Antigen** | **Manufacturer** | **Cat. no.** | **Lot no.** | **Animal** | **Mono or poly** | **Dilution** |
| --- | --- | --- | --- | --- | --- | --- |
| alpha-2-HS-glycoprotein | Abcam | ab34505 | 343490 | goat | poly | 1/10,000 |
| Apolipoprotein A-I | Academy Bio  Medical Company | 11A-G2b | 032860-3 | goat | poly | 1/5,000 |
| Complement C3 | SantaCruz | sc-28294 | D1805 | mouse | mono | 1/500 |
| Complement C4-A | SantaCruz | sc-74524 | L2107 | mouse | mono | 1/200 |
| Gelsolin | Abcam | ab11081 | 468410 | mouse | mono | 1/2,000 |
| Haptoglobin | Genway Biotech | 15-288-20080 | 04GA0023-040116 | chicken | poly | 1/5,000 |
| alpha-1-acid glycoprotein 1 | SantaCruz | sc-69753 | F2807 | mouse | mono | 1/100 |
| alpha-1-antitrypsin | Abcam | ab9400 | 554299 | mouse | mono | 1/4,000 |
| alpha-1-antichymotrypsin | SantaCruz | sc-22747 | A0204 | rabbit | poly | 1/500 |

HRP-conjugated 2nd antibodies used were: rabbit anti-goat IgG-HRP (KPL 14-13-06; lot 08183) at 1/5,000 dilution; goat anti-rabbit IgG-HRP (KPL 074-1516; lot 090184) at 1/10,000 dilution; goat anti-mouse IgG-HTP (KPL 074-1802; lot 060744) at 1/10,000 dilution; rabbit anti-chicken IgY-HRP (Chemicon, AP162P; lot 0510012472) at 1/25,000 dilution.
